# Enhanced Intrusion Detection with Data Stream Classification and Concept Drift Guided by the Incremental Learning Genetic Programming Combiner

**DOI:** 10.3390/s23073736

**Published:** 2023-04-04

**Authors:** Methaq A. Shyaa, Zurinahni Zainol, Rosni Abdullah, Mohammed Anbar, Laith Alzubaidi, José Santamaría

**Affiliations:** 1 School of Computer Sciences, Universiti Sains Malaysia, USM, Gelugor 11800, Pulau Penang, Malaysia; methaqabdullah@student.usm.my (M.A.S.);; 2 National Advanced IPv6 Centre (NAv6), Universiti Sains Malaysia, USM, Gelugor 11800, Pulau Penang, Malaysia; 3 School of Mechanical, Medical, and Process Engineering, Queensland University of Technology, Brisbane, QLD 4000, Australia; 4 Centre for Data Science, Queensland University of Technology, Brisbane, QLD 4000, Australia; 5 Department of Computer Science, University of Jaén, 23071 Jaén, Spain

**Keywords:** stream data classification, concept drift, incremental learning, genetic programming combiner, intrusion detection, transfer learning

## Abstract

Concept drift (CD) in data streaming scenarios such as networking intrusion detection systems (IDS) refers to the change in the statistical distribution of the data over time. There are five principal variants related to CD: incremental, gradual, recurrent, sudden, and blip. Genetic programming combiner (GPC) classification is an effective core candidate for data stream classification for IDS. However, its basic structure relies on the usage of traditional static machine learning models that receive onetime training, limiting its ability to handle CD. To address this issue, we propose an extended variant of the GPC using three main components. First, we replace existing classifiers with alternatives: online sequential extreme learning machine (OSELM), feature adaptive OSELM (FA-OSELM), and knowledge preservation OSELM (KP-OSELM). Second, we add two new components to the GPC, specifically, a data balancing and a classifier update. Third, the coordination between the sub-models produces three novel variants of the GPC: GPC-KOS for KA-OSELM; GPC-FOS for FA-OSELM; and GPC-OS for OSELM. This article presents the first data stream-based classification framework that provides novel strategies for handling CD variants. The experimental results demonstrate that both GPC-KOS and GPC-FOS outperform the traditional GPC and other state-of-the-art methods, and the transfer learning and memory features contribute to the effective handling of most types of CD. Moreover, the application of our incremental variants on real-world datasets (KDD Cup ‘99, CICIDS-2017, CSE-CIC-IDS-2018, and ISCX ‘12) demonstrate improved performance (GPC-FOS in connection with CSE-CIC-IDS-2018 and CICIDS-2017; GPC-KOS in connection with ISCX2012 and KDD Cup ‘99), with maximum accuracy rates of 100% and 98% by GPC-KOS and GPC-FOS, respectively. Additionally, our GPC variants do not show superior performance in handling blip drift.

## 1. Introduction

In recent years, all professions and trades have continued to generate large amounts of data, thanks to the rapid development of big data (BD), Internet of Things (IoT) technology, and artificial intelligence (AI) [1]. Data streams, such as network data, weather forecast data, wireless sensor data, and financial and grid data, among others, are being utilised extensively [2]. Data stream mining in particular has a wide range of applications, such as fraud detection [3], spam filtering [4], intrusion detection [2], stock prediction [5], user interest prediction [6], and other applications for modern society [7]. Data stream classification has become a critical learning topic in recent years. High speed, nonstationary data distribution and limitless duration are characteristics of most of these data [8]. Furthermore, the rapid emergence of Industry 4.0 and Industry 5.0, which embed various sub-technologies, including IoT [9], AI [10], and robotics [11], has impelled researchers to focus on addressing data stream classification, including its unique issues and challenges [12].

With network attacks and cybercrime posing an ever-intensifying threat to consumers, businesses, and governments, intrusion detection systems (IDS) have become a crucial aspect of network security [2]. These solutions aid in identifying and thwarting hostile activities while safeguarding information systems, sensitive data, and assets. An IDS employs ongoing analysis of various types of data logs, including network traffic and application/system logs, to detect suspicious activity and unauthorised access attempts on a computer system or network. Specifically, network traffic logs can be considered streaming data, and an IDS can be viewed as a smart learning agent that uses historical attack data to predict and classify future ones [13].

Numerous investigations have demonstrated promising outcomes in the field of IDS through the application of machine learning (ML) and deep learning techniques [14]. However, many of these studies have relied on static data sources, failing to take into account the dynamic nature of the technology [15]. Traditionally, ML focuses on using unvarying data that adequately represent the underlying distribution; however, in actuality, modern problems often do not conform to such simplified models. Real-world applications, such as IDS, often involve data distributions that change over time, leading to the problem of non-stationary learning or concept drift (CD) [16]. This requires adaptive learning techniques that can handle evolving data and consistently maintain high classification accuracy.

**Figure 1:**
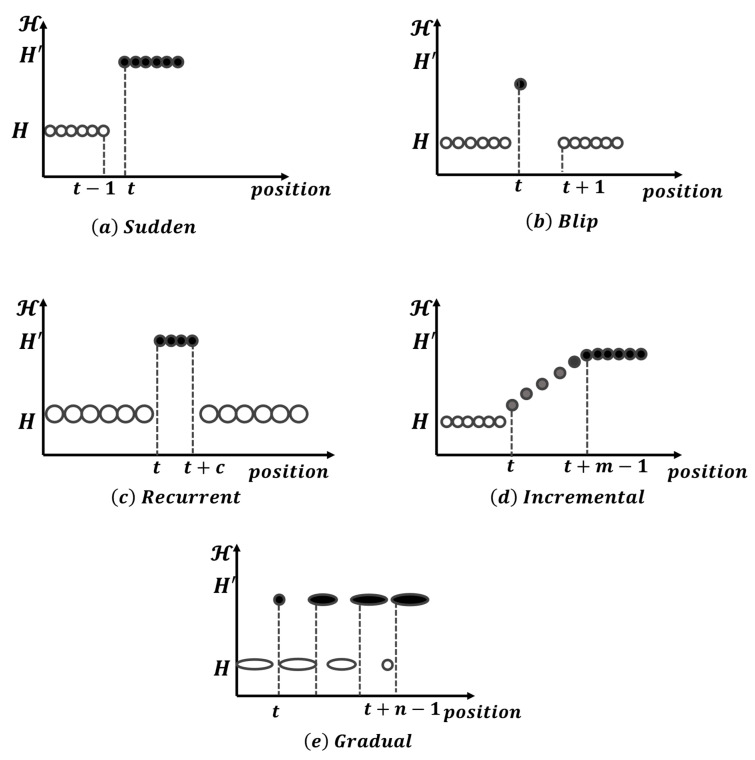
Conceptual diagram of the main variants of concept drift: (**a**) sudden, (**b**) blip, (**c**) recurrent, (**d**) incremental, and (**e**) gradual [19].

The issue of CD, or the gradual change in data characteristics, is common in data stream-based learning problems, including IDS [17]. The dynamic nature of the target concepts can negatively impact the performance of classifiers trained on past data. To address this issue, classifiers must be adjusted to adapt to the changing conditions [18]. Various forms of CD, including sudden, gradual, incremental, recurrent, and blip, are illustrated in Figure 1 [19].

Furthermore, real-world applications are often characterised by the presence of multiple types of CD, which can limit the effectiveness of existing approaches that are designed to address only one type of change [20]. Hence, developing strategies to address CD is an urgent research topic.

In addition to CD, class imbalance is another challenge in the field of data stream classification in general and IDS in particular [21]. Class imbalance refers to the situation where instances in the positive or minority class are significantly underrepresented compared to instances in the negative or majority class. This creates a bias towards the majority class, which traditional classifiers cannot handle. Ensemble methods are a popular approach in ML and can be used to address class imbalance, among other problems [22]. Furthermore, they have proven to be highly effective for data streams because of their accuracy-oriented design, leading to improved predictive performance, generalisation capabilities, and robustness. Thus, ensembles offer unique ways to manage CD and class imbalance [23].

The goal of our contribution is to propose a novel framework for IDS with the capability to handle the majority of variations of CD. It uses ensemble learning based on the Genetic Programming Combiner [24] to deal with both CD and class imbalance issues in IDS. Our proposed framework features the embedding of incremental learning variants of classifiers by providing three options to the user: gradual learning, preserving previous knowledge when features change, and restoring previously gained knowledge when its corresponding features are active again. Hence, the proposed framework is suitable for sudden, incremental, gradual, recurrent, and combined variations of CD. Our key contributions are as follows:

- To the best of our knowledge, this paper provides the first data stream-based classification framework that addresses CD variants.
- It provides various novel strategies for handling CD, such as using an ensemble learning-based GPC, incremental learning using online sequential extreme learning machine (OSELM), and transfer learning (TL).
- It proposes the usage of knowledge preservation OSELM (KP-OSELM) to contend with certain variants of drift that require restoring previous memory, such as sudden, recurrent, and gradual.
- Two novel components are added to the GPC: data balancing and classifiers update. Additionally, a specific coordination between the various sub-models in an incremental GPC is proposed.
- It provides an evaluation of two types of data: synthetic data and real-world data. The real-world data belong to more than one field, with a focus on network IDS. The evaluation includes a comparison against several state-of-the-art methods and the usage of time series classification.

The phenomenon of CD in data streaming scenarios, such as IDS, occurs when the statistical distribution of the data changes over time. This can impact the accuracy of IDS, resulting in false alarms or missed attacks.

To address this challenge, this work proposes an extended variant of the GPC classification method, utilising incremental learning to adapt to changes in the statistical distribution of the data. By replacing existing classifiers with alternatives, adding two new components to the GPC (data balancing and classifiers update) and coordinating between the sub-models, a framework is developed to handle different variants of CD in IDS.

This paper emphasises the significance of addressing CD in IDS to enhance cybersecurity. The utilisation of incremental learning is shown to achieve more accurate and effective detection of security breaches, highlighting the potential for future research in this area.

The paper is structured as follows: Section 2 reviews state-of-the-art methods currently being utilised. Section 3 provides background information, and Section 4 outlines the methodology used in creating the proposed framework. Next, Section 5 presents the experimental work and results. Section 6 highlights the advantages of the developed incremental GPC, and finally, Section 7 draws several conclusions and presents future work.

## 2. Literature Survey

The literature review is divided into two subsections. The first subsection reviews the methods that have been developed for IDS to detect and handle CD. The second subsection examines the methods that have focused on dealing with different variants of CD, regardless of the application area.

### 2.1. CD-Aware IDS Methods

One approach proposed in [24] is an ensemble-based framework for online IDS, where the ensemble is updated through an incremental stream-oriented learning scheme. This approach uses genetic programming (GP) to derive a combiner function and is supported by a system architecture that integrates drift detection and adaptation. However, this approach lacks support for incremental learning, which may affect its ability to adapt to certain variants of drift, such as gradual drift.

In [2], the researchers developed a distributed ML-based ensemble technique to detect the presence of CD in network traffic and detect network-based attacks. The work is divided into three parts: using Random Forest and Logistic Regression as level zero learners, adopting Support Vector Machine (SVM) as a level one learner, and introducing sliding window-based K-means clustering. However, the performance of their approach using different variants of CD is not reported.

Another approach proposed in [17] is INSOMNIA, an intrusion handling method that continuously updates the underlying ML model when CD occurs. Both active learning and label estimation are used to reduce latency in model updates and labelling overhead, respectively. Additionally, an explainable AI is used to better interpret how the model reacts to shifting distributions. However, this approach does not handle the recurrent, blip, and combined variants of CD.

In [25], the authors proposed an approach for CD detection in real-world security applications operating under realistic threat models. They used an embedding network with a contrastive loss objective to capture the similarity/dissimilarity of samples and a novel distance function that captures both instance and class-level fidelity to train an NCM classifier. However, their approach does not focus on adaptation and fails at detecting incremental drift.

In [20], the authors proposed an adaptive approach for online IDS that uses stream-oriented learning to adapt to CD in real-world environments. Specifically, they utilised an Adaptive Random Forest classifier with an Adaptive Windowing (ADWIN) change detector to detect changes in the data stream and adapt to drift detection. However, the authors did not evaluate the performance of this method on different types of CD.

The authors of [13] conducted a comparison of three online heterogeneous ensembles for IDS in terms of their response to CD, performance accuracy, and run-time complexity. The results indicated that the ensemble comprising an Adaptive Random Forest of Hoeffding Trees combined with a Hoeffding Adaptive Tree had the best performance in handling CD. However, the study did not evaluate the performance of these ensembles on various types of CD.

In [26], a hyper-heuristic framework was proposed to handle CD in IDS, which included genetic optimisation, neural networks, and online-offline stream clustering. However, the performance of this approach was not evaluated with respect to CD variants.

The study described in [1] proposed an approach to predicting CD in real time for a data stream of social network attacks. The method is based on online learning algorithms and employs a fusion of experts to identify anomalies in the data stream. This approach is distinct from previous research in the field and offers a novel solution for addressing the challenge of detecting CD in social network attack data streams.

In [27], a novel intrusion detection model based on reinforcement learning was proposed. The model is designed to operate for extended periods without frequent updates and consists of two strategies. The first strategy uses an ML scheme as a reinforcement learning task to achieve long-term learning, which results in high reliability and classification accuracy over time. The second strategy involves model updates using TL and a sliding window mechanism, which minimises the need for computational resources and human intervention. This approach enables the model to adapt to changing environments and learn from new data without compromising its accuracy.

In [28], a technique was proposed for detecting Botnet cyberattacks using a dynamic sliding window based on residual projection. The technique utilises CD analysis and dynamically updates the sample number by comparing anomalies during the process of finding concepts in data streams.

The authors of [29] presented a multistage automated network analytics framework for adapting to CD in industrial IoT systems. The proposed framework consists of four stages: dynamic data pre-processing, a drift-based dynamic feature selection method, dynamic model learning and selection, and a window-based weighted probability averaging ensemble model. The framework aims to effectively adapt to changing conditions in industrial IoT systems by dynamically selecting relevant features and models based on CD analysis. The study presented in [30] introduced a drift detection technique that utilises the principal component analysis (PCA) method to monitor changes in the variance of features in intrusion detection data streams. To counter these drifts, an online deep neural network (DNN) that dynamically adjusts the sizes of the hidden layers based on the Hedge weighting mechanism was discussed, thus enabling the model to steadily learn and adapt as new intrusion data emerge.

Table 1 provides an overview of existing models in the literature for data stream prediction.

### 2.2. CD-Aware Methods

The detection and management of CD in a data stream has been addressed in a variety of ways. A hybrid block-based ensemble was presented in [31] by means of a framework for multi-class classification in dynamic data streams. In order to respond to various forms of drift, the multi-class framework intends to integrate the key benefits of an online drift detector for a k-class problem and the idea of block-based weighting. However, the strategy has not been tested against every kind of notion drift, and it has not been evaluated against other adoption strategies.

An incremental–decremental SVM CD model with an adjustable shifting window (AIDSVM) was suggested in [32]. It has two key benefits: it is faster than a standard SVM since there is no need for retraining because the model is adjusted on the fly, and it is more accurate because irrelevant samples are eliminated at the right time based on the Hoeffding test. However, the incremental solution is often built more slowly.

Recurrent Adaptive Classifier Ensemble (RACE), a developing method, was suggested in [33] to handle repeating notions. The algorithm employs a drift detector to check whether a drift has occurred. According to the results, the algorithm’s prediction accuracy for nonstationary time series data is improved by knowledge transfer and drift detection. However, the RACE algorithm can be computationally expensive because it needs a substantial amount of memory for storage of all the extremely diverse classes and for storage needs during concept transfer. The ensemble’s utility with nonstationary series data, where a classification delay might be expensive, is further compromised since the concept transfer procedure takes longer and therefore slows the convergence to recurrent ideas.

Probabilistic Exact Adaptive Random Forest with Lossy Counting (PEARL) was proposed in [34]. It was developed to replace drifting trees with pertinent trees previously constructed using both an accurate method and a probabilistic graphical model with Lossy Counting. The precise method uses pattern matching to identify the collection of drifting trees that co-occurred in previous forecasts. The replacement of trees among recurrent CDs is being captured by a probabilistic graphical model that is simultaneously being developed. When the graphical model is stable, it takes the role of the precise method and uses a probabilistic search to identify pertinent trees. Additionally, Lossy Counting is used to offer an additional theoretical assurance for both error rate and space complexity to the graphical model. However, because of the absence of ensemble learning, the system runs the danger of bias, and it ignores some categories of notion drifts.

A system named Nacre was introduced in [35], which can carry out proactive drift detection and live updates to enable seamless adaptation of idea drifts. A drift coordinator foresees the following drift point and evaluates the entering idea. In the end, this will improve classification performance accuracy.

Diversity was suggested as a framework in [36] to deal with various sorts of drift. The framework employs clustering in the model space in an original way to create a varied ensemble and spot recurring ideas. The ensuing variety also hastens adaptation to various forms of drift in which a new notion resembles an earlier concept. However, the framework has been criticised for containing no provision for balanced learning.

The heterogeneous adaptive ensemble model for the categorisation of data streams was presented in [8]. It utilises a dynamic class weighting system and a strategy to keep the ensemble members’ variety. A heterogeneous set of basic learners (Naive Bayes, k-NN, and decision trees) make up the model, which has an adaptive mechanism that considers both the ensemble’s diversity and the members’ individual performances. However, it has been faulted for neglecting some types of conceptual deviations.

Kappa Updated Ensemble (KUE) is an ensemble approach proposed in [37] that combines online and block-based techniques for handling CD, utilising the Kappa statistic to dynamically weight and select base classifiers. To increase diversity, each base learner is trained on a separate subset of characteristics and updated with fresh examples with a specific probability following a Poisson distribution. New classifiers are only included in the ensemble if they significantly improve its quality, and each base classifier can abstain from voting, enhancing the algorithm’s robustness. However, KUE has been tested only for frequent drift scenarios.

Broad Ensemble Learning System (BELS), a stream classification method based on the original BELS, introduced in [38], leverages a dynamic output ensemble layer to overcome its limitations. However, it cannot operate with semi-supervised learning. In [39], Performance Weighted Probability Averaging Ensemble (PWPAE) was proposed for analysing data streams in the IoT. This option is distinctive because it assigns dynamic weights to base learners based on their current performance, rather than using predetermined or fixed weights. The algorithm uses ADWIN and DDM to detect drift. In their study [40], the authors proposed the AdIter method for adapting to concept drift quickly. AdIter is based on GBDT, which helps the GBDT model in selecting the optimal strategy for adapting, whether it be through retraining or tuning. Table 2 summarises the techniques based on the classifier, application, method, drift detection technique, and variant of CD assessed.

### 2.3. Literature Gap

The literature review indicates that while current methodologies for CD-aware IDS have considered knowledge updating and detection, they fail to account for various types of CD and lack evaluation of their strategies in relation to these types. Recent strategies for addressing CD in a data stream have been developed, but none have been assessed in terms of all possible variants. Incorporating memory into the model is essential for handling sudden and recurrent CD, as prior knowledge is required for the future. Given the importance of CD variation, developing a ML model capable of not only detecting CD but also managing it remains a research gap that has yet to be addressed.

## 3. Background

This section provides background information on three classifiers that are utilised in the proposed method: OSELM, feature adaptive OSELM (FA-OSELM), and KP-OSELM. It also gives an overview of the baseline method, GPC, which is used in the study.

### 3.1. Background of OSELM, FA-OSELM and KP-OSELM

#### 3.1.1. OSELM Review

In many applications, data are continuously generated over time rather than available in advance. As a result, it becomes necessary to train on the data block each time new data become available. In [41], OSELM, a mathematical method for conducting online sequential learning for ELM, was developed, consisting of two major phases. In the boosting phase, the primitive ELM method and a few batches of training data are used to train single hidden layer feed-forward networks (SLFNs). After the boosting phase, the boosting training data are discarded, and the OSELM learns the training data one by one or chunk by chunk. Once the learning procedure is complete, all the training data are discarded. The OSELM algorithm’s process is outlined in Algorithm 1, and the symbols used are explained in Table 3.

*H*_0_= [*h_1_*_,_…,*h_Ñ_*]*^T^*, Where:    *h_i_ = [g (W_1_. X_i_ + b_1_)*,…, *g (W_Ñ_. X_i_ + b_Ñ_)]^T^, i =* 1,…, (1)

*h*_(*k*+1)_ = *[g(W_1_ − X_i_ + b_1_),…, g (W_Ñ_. X_i_ + b_Ñ_)]^T^* (2)

(3) $$M_{k+1}=M_{k}-\frac{M_{k}h_{k}+h_{k+1}^{T}M_{k}}{1+h_{k+1}^{T}M_{k}h_{k}+1}$$

(4) $$\beta^{\left(k+1\right)}=\beta^{\left(k\right)}+M_{k+1}h_{k+1}\left(t_{i}^{T}-h_{k+1}^{T}\beta^{\left(k\right)}\right)$$

**Algorithm 1.** The pseudocode of the ELM (OS-ELM) algorithm.**Input:** (1) ℵ = *{(X_i_, t_i_)|X_i_*
∈
*R^n^, t_i_*
∈
*R^m^, i = 1,... , Ñ}* **Output:** trained SLFN **Start Algorithm** 1: Boosting Phase: 2: Assign arbitrary input weight $W_{i}$ and bias $b_{i}$ or center $\mu_{i}$ and impact width $\sigma_{i\;},$ i=1,…,N. 3: Calculate the initial hidden layer output matrix in Equation (1) 4: Estimate the initial output weight β^(0)^ = *M_0_*$H_{0}^{T}$*T*_0_, Where M_0_ = ($H_{0}^{T}$*H*_0_)^−1^ and T_0_ = [t_1,_...,t*_Ñ_*]*^T^.* 5: Set k=0. 6: Sequential Learning Phase: 7: For each further coming observation (*X_i_, t_i_*), where, *X_i_*
∈
*R^n^, t_i_*
∈*; R^m^* and i = *Ñ* + 1, *Ñ* + 2, *Ñ* + 3,…, do 8:  Calculate the hidden layer output vector in Equation (2) 9:  Calculate latest output weight β^(*k+*1)^ based on RLS Equation (3) 10:   Set k=k+1. 11: end 12: **End Algorithm**

#### 3.1.2. FA-OSELM Review

In [42], FA-OSELM was developed as a model that transfers weight values from the previous classifier to the new one. In essence, it is a technique for transferring prior knowledge from a pre-existing neural network to a new one, which considers the difference in the number of features between them. Since the number of hidden nodes (*L*) remains the same in both networks, FA-OSELM provides an input-weight supplement vector $Q_{i}$ and an input-weight transfer matrix P. These allow for the transition from the old weights $a_{i}$ to the new weights ai, with consideration for the equation that accounts for the change in the number of features from $m_{t}$ to $m_{t+1}$:

(5) $$m_{t+1}=\{\;{w^{\prime }}_{i}=w_{i}.P+Q_{i}{\}}_{i=1}^{L}$$

(6) $$P={\left[\begin{array}{ccc} P_{11} & \cdots & P_{1m_{t+1}}\\ \vdots & \ddots & \vdots \\ P_{m_{t}1} & \cdots & P_{m_{t}m_{t+1}} \end{array}\right]}_{m_{t}\times m_{t+1}}$$

*Q_i_ = [ Q_1_... Q_mt+1_ ]1×m_t+1_* (7)

where matrix *P* must adhere to the following rules:

- For every line, there is a single ‘1’; the rest of the values are all ‘0’.
- Every column has no more than a single ‘1’; the rest of the values are all ‘0’.
- $P_{ij}=1$ signifies that following a change in the feature dimension, the ith dimension of the original feature vector will become the jth dimension of the new feature vector.
- When the feature dimension increases, $Q_{i}$ will function as the supplement. It also adds the corresponding input weight for the newly added attributes. Furthermore, the rules below apply to $Q_{i}$.
- Lower feature dimensions indicate that $Q_{i}$ can be termed as an all-zero vector. Hence, no additional corresponding input weight is required by the newly added features.
- In cases where the feature dimension increases, if the new feature is embodied by the ith item of ${w^{\prime }}_{i}$, a random generation of the ith item of $Q_{i}$ should be carried out based on the distribution of $w_{i}$.

#### 3.1.3. KP-OSELM Review

In [43], KP-OSELM was introduced, which is a modified version of OSELM that aims to retain the weights of inactive features and utilise them when they become active. The learning equations of this method closely resemble those of classical OSELM but with the use of tensing as the objective function and changes to the constraint of zero features within one data chunk.

The boosting equations, shown in Equations (7)–(10), and the iterated equations, shown in Equations (11)–(14), differ from those of the classical OSELM equations [44], in terms of input data. In KP-OSELM, the input vector *X_i_* is not utilised; instead, it is replaced with vector $\ddot{X}$*_i_*, which is computed using Equation (12). This equation shows that vector $X_{i}$ has the identical element as $X_{i}$ for active features, and the non-active features are assigned zeros. I denotes the active features, while F denotes the entire feature set.

(8) $$\beta^{\left(0\right)}\;=\;M_{0}H_{0}^{T}y_{0}$$

(9) $$M_{0}={\left(H_{0}^{T}H_{0}\right)}^{-1}$$

(10) $$y_{0}={\left[y_{1},\ldots ,y_{N^{k}}\right]}^{T}$$

(11) $$H_{0}={\left[h_{1,\ldots ,}h_{N^{k}}\right]}^{T}$$

(12) $$h_{i}=[\mathrm{tansig}\;(W_{1}.\;{\ddot{X}}_{i}+b_{1}),\;\ldots ,\;\mathrm{tansig}(W_{\tilde{N}}.{{\ddot{X}}_{i}+b_{\tilde{N}}),]}^{T},\;i=1,\ldots ,N^{k}.$$

(13) $${\ddot{X}}_{i}=\left({\ddot{x}}_{j}\right)\;{\ddot{x}}_{j}=\left\{\begin{array}{c} x_{j};j\in I\\ 0;\;\mathrm{otherwise}\; \end{array}\right.\;I=\left\{i_{1},i_{2},\ldots .i_{k}\right\}\subseteq F=\left\{1,2\ldots ..n\right\}$$

(14) $$\beta^{\left(k+1\right)}=\beta^{\left(k\right)}+M_{k+1}h_{k+1}\left(t_{i}^{T}-h_{k+1}^{T}\beta^{\left(k\right)}\right)$$

(15) $$M_{k+1}=M_{k}-\frac{M_{k}h_{k}+h_{k+1}^{T}M_{k}}{1+h_{k+1}^{T}M_{k}h_{k+1}}$$

Algorithm 2 presents the pseudocode for the learning and prediction phases, which begin with the boosting phase that trains the initial $SLFN_{0}$ network with the boosting data ($D_{0}$, $y_{0}$). The data consist of the RSSI information associated with active features, along with the location of each record. The encode function is utilised to represent non-active features with zero values. The process is repeated for every available data chunk, where each chunk contains an equal number of active features. It should be noted that the training and prediction functions use the same formulas as those used for OSELM, which are given in Equations (2) and (4).
**Algorithm 2.** The pseudocode for training and prediction using KP-OSELM.**Input**: (1) $D_{k}$ (2) ${\mathrm{SLFN}}_{0}$ **Output:** (1) ACC **Start:** 1.: *x*_0_ = Encode (*D*_0_) 2.: SLFN_1_ = OSELMTrain (SLFN_0_, *x*_0_, *y*_0_) 3: for k = 1 unit N 4:                   $x_{k}=\;\mathrm{Encode}\;\left(D_{k}\right)$ 5:                  $Y_{k}=\mathrm{Predict}\left(SLFN_{k},x_{k}\right)$ 6:                $\mathrm{ACC}\;=\;\mathrm{calculateAccuracy}\;\left({\hat{y}}_{k},y_{k}\right)$ 7:  ${\mathrm{SLFN}}_{k+1}=\;\mathrm{OSELMTrain}\;\left({\mathrm{SLFN}}_{k},x_{k},y_{k}\right)$ 8:   end 9: **End Algorithm**

**Figure 2:**
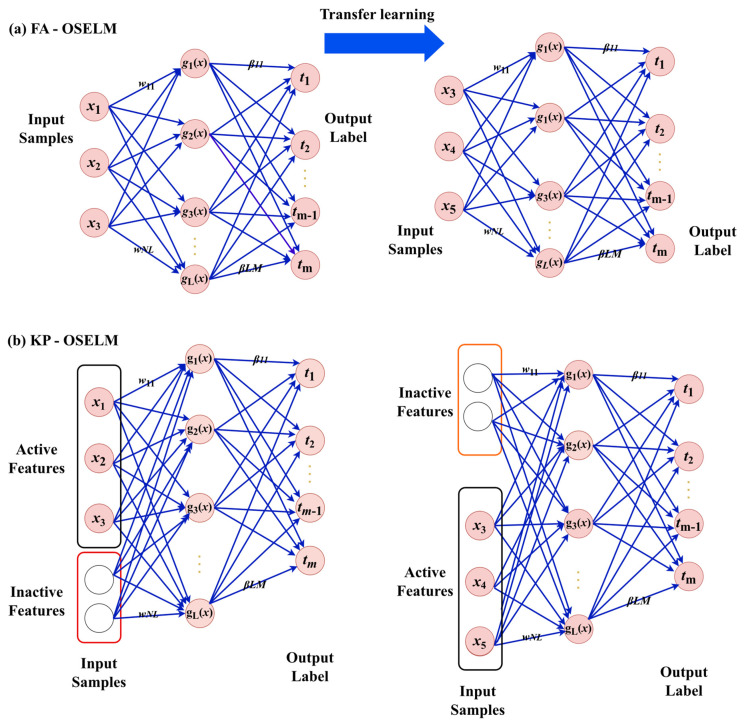
The evolution of a Single Layer Feed-forward Network (SLFN) with respect to the changes in the number of features. The structure of two variants of Online Sequential Extreme Learning Machine (OSELM) algorithms, namely, Feature Adaptive OSELM (FA-OSELM) and Knowledge Preserving OSELM (KP-OSELM), are shown in (**a**) and (**b**), respectively.

Figure 2 illustrates the neural network’s evolution as it moves from one set of active features to another and its relationship with the inputs. It is important to note that KP-OSELM’s structure or topology remains unchanged, unlike FA-OSELM, which updates the input number based on the active features and utilises a separate TL block to transfer the necessary weights from the previous network to the new one, as shown in Figure 2a. In contrast, KP-OSELM retains all inputs, regardless of whether they are active or non-active, as depicted in Figure 2b. However, to ensure that the activation functions pass through (0,0), non-active features are encoded with the same value of zero for the current tansig. The purpose is to eliminate the influence of non-active features in the network’s decision-making process.

### 3.2. GPC

**Figure 3:**
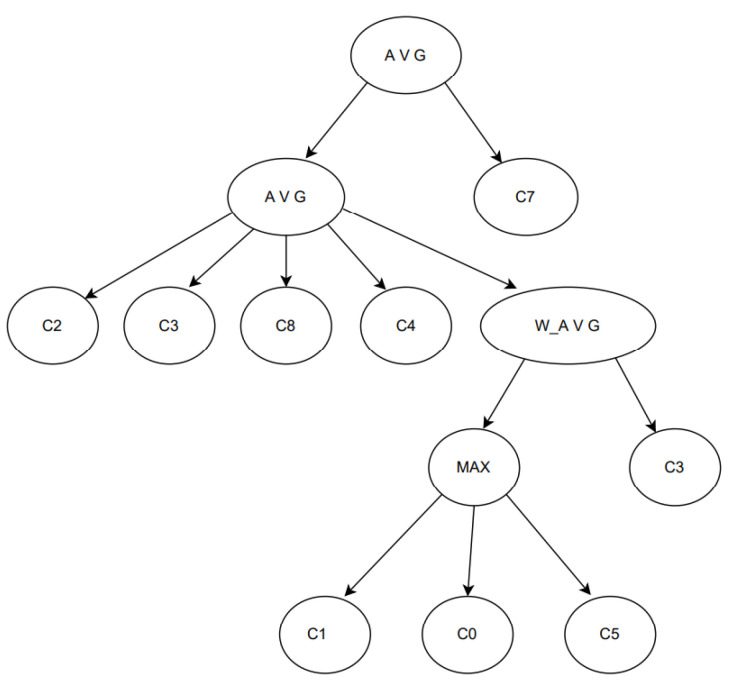
An example of a GPC graph: the nodes represent one classifier or one aggregation operator between one or more classifiers. Some operators are max, average, and weighted average. The classifiers range from $C_{0}$ to $C_{8}$ [24].

The goal of the GPC is to build a graph of classifiers with operators for aggregating between them and to use it for prediction, and GP is the process by which the graph is built. An example of one graph is given in Figure 3.

The pseudocode of the main algorithm is presented in Algorithm 3 of the GPC. It accepts an instance from the data stream x with the corresponding label y, as well as the buffer (designated as buffer), an ensemble of data structure (designated as ensemble), drift detection object (designated as DDO), the maximum number of classifiers in the ensemble (designated as maxC), the number of instances in the buffer to apply model induction after exceeding it, and a Boolean variable that indicates a drift occurrence (designated as drift). In addition, the algorithm takes NumHiddenNeurons and ActivationFunc as inputs. The first one, NumHiddenNeurons, indicates the number of classifier neurons that will be provided to the ensemble, and the second one, ActivationFunc, indicates the types of activation functions used for each classifier. The output of the algorithm is the updated ensemble, buffer, and drift. The algorithm uses the input data to detect the existence of drift, update the ensembles in the case of drift, and return new, updated classifiers and buffer to the next iteration. The first step of the algorithm is to check for the existence of a current ensemble; if the ensemble is still Null, which indicates the initial starting of the algorithm, the algorithm calls the initialisation. The initialisation returns a set of classifiers that will be used randomly for creating the first ensemble using createEnsemble. The second step of the algorithm is to use the current ensemble to predict the class of x using globalSupportDegree. The ensemble uses an existing graph of classifiers to enable the prediction, then checks whether ground truth is available. If the ground truth is available, it adds the sample to the buffer and enables drift detection. In the case of drift, the ensemble is updated. First, the addExamplesFromAnotherClass procedure is called, which is responsible for balancing the buffer. Then, the existing models in the ensemble are trained by splitting the data into two subsets, $X_{1}$ and $X_{2}$, training them on the first subset $X_{1}$ and preserving the other subset for use in aggregation in the ensemble. The model induction procedure is then called to find new classifiers to be inserted into the current base classifier. The third step the algorithm calls baseClassifierUpdate. In the fourth step the algorithm checks the current length of the ensemble to see whether it exceeds maxC. If it does, the model replacement is activated, and the genetic programming algorithm is executed. On the other hand, if no drift is detected, the algorithm clears the buffer to update it with data from the next chunk to keep the models up to date.
**Algorithm 3.** Pseudocode of GPC.**Input:** (1) x: an instance from the data stream (2) y: the corresponding label of x (3) buffer: a list of instances (4) ensemble: an ensemble data structure (5) DDO: a drift detection object (6) maxC: the maximum number of classifiers in the ensemble (7) n: the number of instances in the buffer to apply model induction after exceeding it (8) drift: a Boolean variable indicating a drift occurrence (9) NumHiddenNeurons, (10) ActivationFunc **Output:** (1) ensemble: the updated ensemble (2) buffer: the updated buffer (3) drift: the updated drift flag Start Algorithm 1: If (ensemble==Null) 2:  classifiers = initialisation(NumHiddenNeurons,ActivationFunc); // Algorithm 4 3:  ensemble = createEnsemble(classifiers); 4: End 5: ypred = globalSupportDegree(ensemble, x)  // Algorithm 5 6: if y! = None then 7:   buffer.append((x, y)) 8:   drift = DDO.detect(x, y! = ypred 9:   if len(buffer) >= n then 10:   **if** drift: 11:     drift = False 12:     buffer = addExamplesFromAnotherClass(buffer) 13:     $X_{1},{\;X}_{2},{\;y}_{1},{\;y}_{2}$ = trainTestSplit(buffer) 14:     newClassifiers = modelInduction($X_{1},{\;y}_{1}$, generateClassifiers()) 15:     ensemble.baseClassifiers = ensemble.baseClassifiers = baseClassifierUpdate(ensemble.baseClassifiers, newClassifiers, X_1, X_2, L = maxC, Key = ‘validScore’) // Algorithm 6 16:   **if** len(ensemble.baseClassifiers) > maxC then 17:   ensemble.baseClassifiers = modelReplacement(ensemble.baseClassifiers, method = ‘best’) //Algorithm 7 18:   ensemble.program = geneticProgramming(ensemble.baseClassifiers, $X_{2},{\;y}_{2}$) ensemble.baseClassifiers = modelReplacement(ensemble.baseClassifiers, method = ‘best’) //Algorithm 7 19:  **End** 20:  Else 21:   buffer.clear() 22:   return ensemble, buffer, drift 23:  End 24: end 25: **End Algorithm**

## 4. Methodology

This section presents the developed methodology. It starts with the problem formulation, and then it presents a novel variant of the GPC. Next, it presents the sub-models in the incremental GPC, i.e., initialisation, classifier update, model replacement, and prediction. Finally, the methodology ends with presenting the evaluation metrics.

### 4.1. Problem Formulation

Considering that we have stream data represented as $\left({\vec{x}}^{1}.\;y^{1}\right).\ldots .\left({\vec{x}}^{t}.y^{t}\right)\;\mathrm{as}\;t\to \infty ,$ where ${\vec{x}}^{t}$ denotes the feature part, $y^{t}$ denotes the label $Y=\cup_{i=1}^{c}\left\{y_{i}\right\}\cup ∆$, ∆ denotes that the instance is not labelled, c denotes the number of classes, and $y_{i}$ denotes the class i, our goal is to build a prediction model that associates features to their label, or $h:\vec{x}\to y$. The prediction model uses relevant features for this purpose and ignores the irrelevant features. At the same time, the classifier should be adapted to the added dynamic on the data in terms of CD variants. The prediction should minimise the error that is calculated as $e=\overset{t}{\underset{k=1}{\sum }}\left(y_{k}-{\tilde{y}}_{k}\right),$ where ${\tilde{y}}_{k}$ denotes the predicted sample k.

### 4.2. Incremental Learning GPC

The framework comprises several components. First, there is an interface that enables the user to choose between three operating modes: OSELM, FA-OSELM, and KP-OSELM. Each operating mode is associated with a specific type of classifier. The OSELM classifier is incremental but lacks learning transfer or memory of weights for previously active features. The FA-OSELM classifier is also incremental, but it includes a feature-adaptive model that allows for transferring the weights when there is a change in the number of features. The KP-OSELM classifier is built based on the maximum number of features in order to preserve the weights of non-active features for future use.

The input to the framework is streaming data that passes through a data balancing block, which updates non-balanced data with previously stored data from minority classes. The classifier is updated with each new chunk of data that arrives. A drift detector is responsible for deciding whether to update the graph of the GPC by using model update and replacement, which is necessary because drift occurrence is the only reason for using a significant update of the core of the classifiers. Finally, the GPC prediction represents the last phase of the framework for producing the result.

Incremental learning is an ML technique that enables a system to learn continuously from new data, updating its knowledge and predictions in real time as new information becomes available. In the context of the GPC framework, incremental learning is used to update the classifiers with each new chunk of data that arrives. This approach allows the framework to adapt to changes in the data distribution and learn from new patterns and trends that may emerge over time.

The incremental learning approach used in the GPC framework provides several benefits. First, it allows for efficient and scalable processing of large streams of data, which can be particularly significant in applications that require real-time processing and in which data are received at high rates. Second, it enables the system to adapt to changes in the data distribution and learn from new patterns and trends, improving the accuracy and robustness of the predictions. Finally, incremental learning can help to reduce the impact of CD by allowing the system to continuously update its models and adapt to the new data distribution.

**Figure 4:**
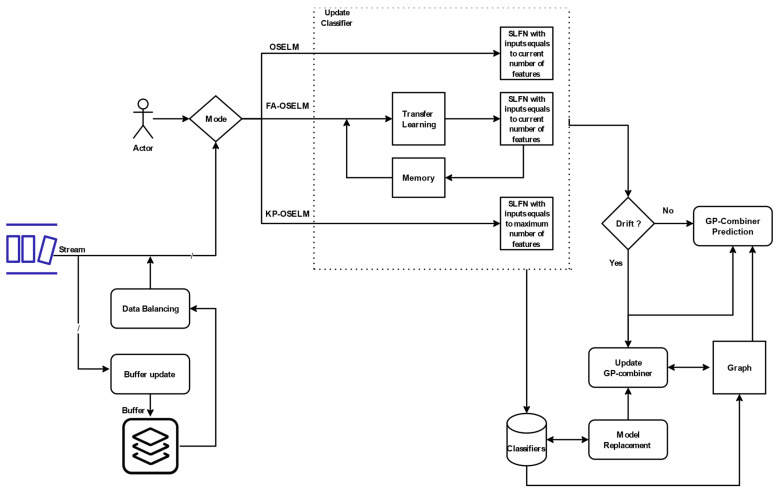
Framework of the incremental GPC using three modes: OSELM, FA-OSELM, and KA-OSELM.

The incremental learning GPC is depicted in Figure 4. As shown, the user provides the mode of operation for the GPC (OSELM, FA-OSELM, or KP-OSLEM). After setting one of the three modes, the GPC is designated with a suffix according to the type of classifier. For OSELM, the GPC is designated as GPC-OS. For FA-OSELM, the incremental GPC is designated as GPC-FA. For KP-OSELM, the GPC is designated as GPC-KP. The classifiers are updated according to the mode set by the user. For OSELM, the classifiers are updated incrementally, and for FA-OSELM and KP-OSELM, the classifiers are updated incrementally with TL and KP, respectively.

The data are fed into the incremental GPC as stream data, combined of multiple chunks organised sequentially. The first stage after feeding the data is to perform a buffer update. The role of the buffer update is to keep data for balancing the training data when we have minority and majority class to avoid biases. After updating the data, the process of classifier update is performed.

The update of the classifier is an incremental update with the most recent data and is followed by a step of model replacement that upgrades the classifiers in the existing graph. Next, the system checks for the occurrence of drift. If a drift is detected, then the GPC is updated.

### 4.3. Initialisation

The initialisation does not mean initial training of the classifier. Instead, it is simply creating the topologies of the classifiers according to the number of hidden neurons and type of activation function. The classifiers after initialisation will be trained later in the process of data stream update. Hence, the pseudocode for the initialisation algorithm in the incremental GPC is presented in Algorithm 4. This algorithm generates the initial classifiers for the core and takes two configuration inputs: NumHiddenNeurons and ActivationFunc. NumHiddenNeurons is an array that contains different values for the number of hidden neurons for the classifier, while ActivationFunc is an index that represents different values of activation functions. The output of the initialisation algorithm is a set of initial classifiers that will be used in the subsequent phases of the incremental GPC.
**Algorithm 4.** Pseudocode of initialisation of the incremental GPC with classifier.**Input:** (1) NumHiddenNeurons: number of hidden neurons to be used in the classifier (2) ActivationFunc: activation function to be used in the classifier **Output:** (1) classifiers: list of machine learning models to be used in ensemble 1: **Start Algorithm** 2:  classifiers = list() 3:  **for** index = 1 until NumHiddenNeurons 4:    classifiers.Append(OSELM(NumHiddenNeurons(index), ActivationFunc(index))) 5:  **end** 6: Return classifiers 7: **End Algorithm**

### 4.4. Prediction Using Global Support Degree

The last step in the aggregation of classifiers is the decision-making process to provide the final predicted output. The pseudocode is presented in Algorithm 5. The inputs are the baseClassifiers and the sample to be tested, while the output is the decision profile for the tested sample. The algorithm first initialises the decision profile. Next, it goes through the classifiers one by one and provides a vector of prediction probabilities for each sample. Finally, it combines all these vectors into a single decision profile that is properly shaped. The predicted class is the one corresponding to the maximum probability.
**Algorithm 5.** Pseudocode of global support degree for the incremental GPC.**Input:** (1) baseClassifiers: custom data structure to store classifiers (2) sample: new instance from the data **Output:** (1) decisionProfile: support degree, 2D array contains prediction probability of each classifier (2) ypred 1: **Start Algorithm** 2:  decisionProfile = []  // Initialize decision profile list 3:  for each clf in baseClassifiers do  // Iterate over each classifier in the base classifiers 4:   decisionProfile.add(clf.predictProbability(sample)) 5:  end for 6:  decisionProfile = array(decisionProfile, shape = (numOfClassifier, numOfClasses)) 7:  decisionProfile = argmax(decisionProfile.sum(axis = 0)/decisionProfile.shape [0]) 8:  ypred = class of max decisionProfile 9: **End Algorithm**

### 4.5. BaseClassifier Update

The pseudocode of the classifier update is presented in Algorithm 6. It accepts several inputs: First, baseClassifiers, which denotes a custom data structure to store classifiers and their corresponding time of insertion, training accuracy, and validation accuracy. Second, newClassifiers, which denotes a list of classifiers that will be added to the base classifiers. Third, Xtrain, which denotes a set of samples to select the best L models from newClassifiers based on validation accuracy. Fourth, XValid, which denotes a set of samples to evaluate each classifier. Fifth, L, which denotes the number of classifiers that will be added to baseClassifiers. Sixth, the key that includes the method or criteria for favouring classifiers. Seventh, the labelled samples from the buffer that are used to update the knowledge of the classifier incrementally when OSLEM is used and incrementally with memory and TL when FA-OSELM and KP-OSELM are used.

The output of the algorithm is an updated baseClassifier after inserting new classifiers. The algorithm uses one of three criteria for classifier replacement: training accuracy, validation accuracy, or insertion time. After sorting the new classifiers used as input to the algorithm according to one of the three criteria, it selects the first L classifiers and inserts them into the base classifiers.
**Algorithm 6.** Pseudocode of classifier update for the incremental GPC.**Input:** (1) baseClassifiers: list of existing classifiers (2) newClassifiers: list of new classifiers to be added (3) Xtrain: training data (4) XValid: validation data (5) L: maximum number of classifiers to be kept (6) Key: sorting key for the classifiers (7) LabeledSamples: labeled samples for incremental learning **Output:** (1) baseClassifiers: updated list of classifiers 1: **Start Algorithm** 2: for each clf in newClassifiers do 3:  use LabeledSamples to update classifier incrementally for OSELM and with TL for FA-OSELM and KP-OSELM 4:  clf.trainScore = accuracy(clf.predict(Xtrain), ytrain) 5:  clf.validScore = accuracy(clf.predict(XValid), yvalid) 6:  clf.insertionTime = time.currTime() 7:  end 8:  newClassifiers = sort(newClassifiers, key) 9:  newClassifiers = newClassifiers[:L] 10:   for each clf in newClassifiers do 11   baseClassifiers.insert(clf) 12:  end 13: **End Algorithm**

### 4.6. Model Replacement

The pseudocode for model replacement is presented in Algorithm 7. The inputs include the baseClassifiers, which determine the replacement method, and the labelled samples used to update the knowledge of the classifier incrementally. The output is the updated baseClassifier. The replacement strategy is based on several factors, including validation score, random selection, and insertion time. The algorithm sorts the classifiers based on the selected criteria and drops the last third of the classifiers.
**Algorithm 7.** Pseudocode of model replacement for the incremental GPC.**Input:** (1) baseClassifiers: list of existing classifiers (2) method: string that determines the replacement method to be used **Output:** (1) baseClassifiers: updated list of classifiers after replacement 1: **Start Algorithm** 2:  strategy = {‘best’: ‘validScore’, ‘random’: ‘insertionTime’, ‘wheel’: ‘validScore’} 3:  key = strategy[method] 4:  baseClassifiers = sort(baseClassifiers, key = key) 5:  length = len(baseClassifiers) 6:  threshold = length/3 7:  baseClassifiers = baseClassifiers [0:threshold] 8: **End Algorithm**

### 4.7. Evaluation Metrics

For providing the evaluation metrics, assuming that a record is entered to be tested and is predicted to be an attack, then the prediction is P. Otherwise, if the record is predicted to be normal, the prediction is N. Either way, the prediction can be true (T) or false (F). Hence, we have four values for the evaluation, TP, FN, FP, and TN.

#### 4.7.1. Accuracy

Accuracy is measured by the total number of true predictions over the total number of predictions. The equation of the accuracy is presented in Equation (15).

(16) $$\mathrm{ACC}=\frac{\mathrm{TP}+\mathrm{TN}}{P+N}=\frac{\mathrm{TP}+\mathrm{TN}}{\mathrm{TP}+\mathrm{TN}+\mathrm{FP}+\mathrm{FN}}$$

#### 4.7.2. F-Score

F-score represents twice multiplication of the precision and recall divided by the summation. The equation of F1-score is presented in Equation (16).

(17) $$F1-\mathrm{score}\;=\frac{2\times \;\mathrm{recall}\;\times \;\mathrm{precision}\;}{\;\mathrm{recall}\;+\;\mathrm{precision}\;}$$

#### 4.7.3. AUC

Area under the curve (AUC) ranges between 0 and 1 and represents the probability that a random positive example is positioned to the right of a random negative example. A learner with prediction 100% true has an AUC equal to 1, and a learner with prediction 0% true has an AUC of 0.

### 4.8. Datasets

First, according to the literature, there is a shared method that seeks to introduce CDs into a synthetic dataset while evaluating frameworks for data stream classification. The developed approach will be evaluated using synthetic and real-world datasets to compare the performance of IDS.

#### 4.8.1. Synthetic Datasets

Table 4 lists the parameters used for generating synthetic stream data using the Massive Online Analysis (MOA) framework. These parameters included the number of samples, chunk size, test percentage, and number of features for each type of CD.

The number of samples was set to 80,000, which represented the total number of instances in the stream. The chunk size was set to 8000, which represented the number of instances processed in each iteration of the stream. The test percentage was set to 0.8, which meant 80% of the data are used for training, and 20% are used for testing.

The number of features for each type of CD was also specified. The gradual CD had four features, the recurrent drift had 11 features, the blip had 16 features, the incremental drift had 11 features, and the sudden drift had four features.

For generating the data stream, we utilised the Idea Drift Stream generator in MOA. This generator allowed us to add all five types of CD to the stream. The synthetic datasets we created using MOA included a range of attributes that were designed to represent different aspects of real-world data. These attributes were chosen based on their relevance to the task at hand and their ability to simulate different types of CD. For example, the gradual CD was designed to represent slow changes in the underlying data distribution over time, while the sudden drift was designed to represent abrupt changes in the data distribution.

Overall, the use of synthetic datasets generated by MOA provided a useful tool for evaluating and comparing different online learning algorithms in a controlled environment, where the underlying data distribution is known and can be manipulated to simulate different types of CD.

#### 4.8.2. Real-World Datasets

For real-world evaluation, we used four types of real-world datasets: KDD Cup ’99, CICIDS-2017, CSE-CIC-IDS-2018, and ISCX2012.

1. KDD Cup ‘99: Frequently used to test threat detection models based on ML and contains 42 features for evaluating anomaly detection methods [45].
2. CICIDS-2017: Covers benign and up-to-date frequent attacks and contains 80 features [46].
3. CSE-CIC-IDS-2018: Contains 80 features and was employed in 2018, with a data collection period of ten days [46].
4. ISCX2012: Comprises 19 features and is well recognised for its use of ML-based network IDS modelling for evaluating the performance of the models [47].

A statistical overview of real-world datasets is presented in Table 5.

## 5. Experimental Works and Results

The evaluation was performed using two types of datasets: synthetic and real-world. The former contained artificial variants of CDs, while the latter contained actual CDs generated from IDS data.

### 5.1. Synthetic Datasets

The evaluation of the synthetic dataset was performed using two bar graphs that depict the overall performance and time series for presenting the detailed performance. Each of them is presented in one of the two subsequent sections.

#### 5.1.1. Overall Performance

**Figure 5:**
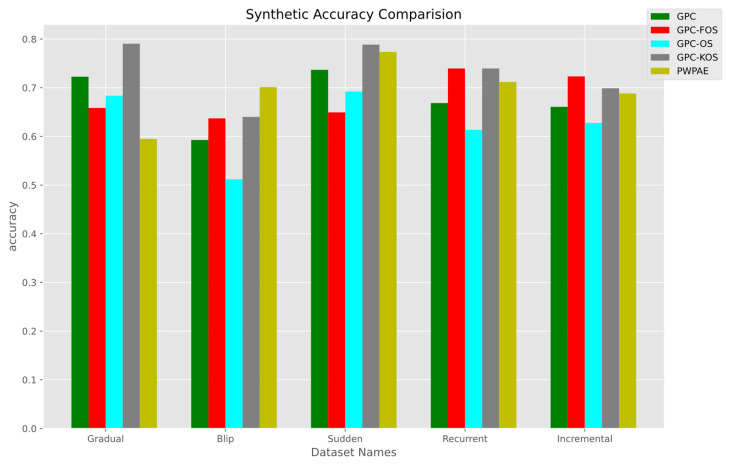
Accuracy of the incremental GPC (OS, FOS, KOS) variants and benchmarks (GPC, PWPAE) for synthetic data.

**Figure 6:**
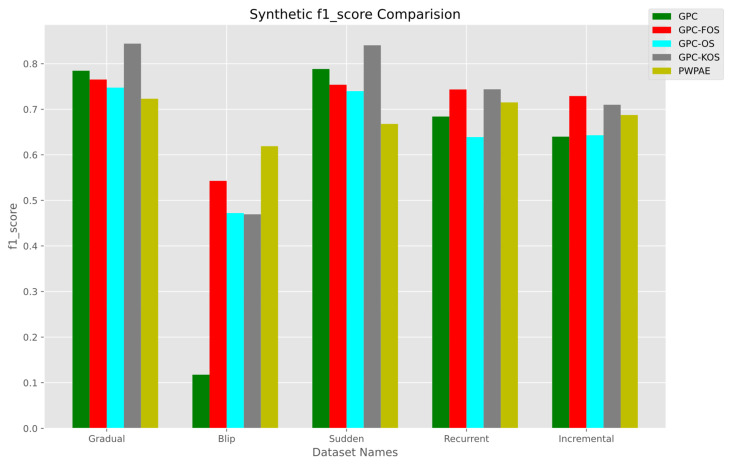
F1-score of the incremental GPC (OS, FOS, KOS) variants and benchmarks (GPC, PWPAE) for synthetic data.

**Figure 7:**
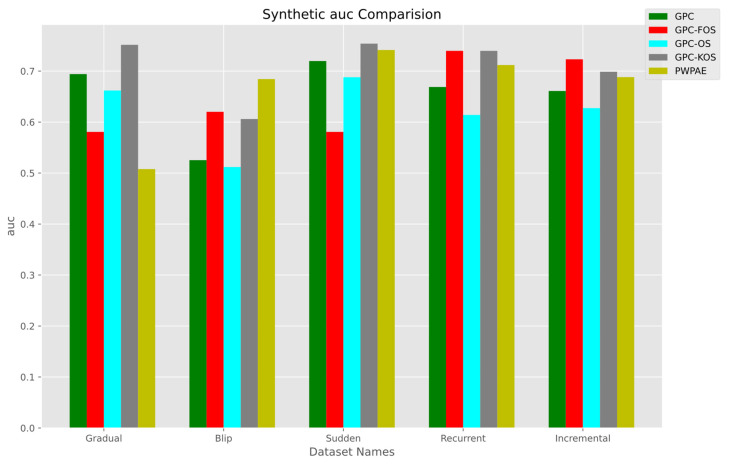
AUC of the incremental GPC (OS, FOS, KOS) variants and benchmarks (GPC, PWPAE) for synthetic data.

The results of the synthetic data analysis using the metrics of accuracy, F1-score, and AUC are presented in Figure 5, Figure 6 and Figure 7. Figure 5 shows that in the gradual scenario, GPC-KOS had the highest accuracy of 0.7905, while PWPAE had the lowest accuracy of 0.594899. In the sudden scenario, GPC-FOS had the lowest accuracy of 0.649300, while GPC-KOS had the highest accuracy of 0.788550. In the recurrent scenario, GPC-FOS and GPC-KOS had the highest accuracy of 0.739600 and 0.739550, respectively, while GPC-OS had the lowest accuracy of 0.613600. In the incremental scenario, GPC-FOS had the highest accuracy of 0.723250, while GPC-OS had the lowest accuracy of 0.627600. In the blip scenario, PWPAE had the highest accuracy of 0.701134, while GPC-OS had the lowest accuracy of 0.511950. Hence, from the results provided, we can see that the accuracy varied across the different types of CD scenarios and GPC variants, but overall, GPC-KOS was superior.

Figure 6 shows that in the gradual scenario, GPC-KOS had the highest F1-score of 0.844099, while PWPAE had the lowest F1-score of 0.723136. In the sudden scenario, GPC-KOS had the highest F1-score of 0.840469, while PWPAE had the lowest F1-score of 0.667857. In the recurrent scenario, GPC-KOS and GPC-FOS both had the highest F1-score of 0.743891, while GPC-OS had the lowest F1-score of 0.638946. In the incremental scenario, GPC-FOS had the highest F1-score of 0.7298088, while the GPC had the lowest F1-score of 0.639919. In the blip scenario, PWPAE had the highest F1-score of 0.618994, while the GPC had the lowest F1-score of 0.117647.

Figure 7 shows that in the gradual scenario, GPC-KOS had the highest AUC of 0.751566, while PWPAE had the lowest AUC of 0.507830. In the sudden scenario, GPC-KOS had the highest AUC of 0.753872, while GPC-FOS had the lowest AUC of 0.580746. In the recurrent scenario, GPC-FOS and GPC-KOS had the highest AUC of 0.739728, 0.739689, respectively, while GPC-OS had the lowest AUC of 0.614074. In the incremental scenario, GPC-FOS had the highest AUC of 0.723171, while GPC-OS had the lowest AUC of 0.627427. In the blip scenario, PWPAE had the highest AUC of 0.684335, while GPC-OS had the lowest AUC of 0.511852.

Based on the analysis of the numerical values of accuracy, F1-score, and AUC for GPC variants (OS, FOS, KOS) and benchmarks in synthetic data, it appeared that GPC-KOS performed consistently well across all four types of data distributions. It achieved the highest accuracy and F1-score for the gradual and sudden distributions, the highest F1-score for the recurrent distribution, and the highest accuracy and F1-score for the incremental distribution. While GPC-FOS and GPC-OS performed well in some cases, they were not as consistent as GPC-KOS. Overall, GPC-KOS appeared to be the GPC variant that performed the best for synthetic data classification tasks.

We present a summary of the numerical values of accuracy, F1-score, and AUC for each of our developed models and the benchmarks in Table 6. It is evident that the superior model was GPC-KOS, as it outperformed PWPAE in gradual and sudden drift and GPC-OS and the GPC in recurrent and incremental drift, respectively. On the other hand, PWPAE performed better only in the blip type of drift.

#### 5.1.2. Time Series

We provide a detailed analysis of the performance of each model by analysing the time series behaviour of each measure. Furthermore, we provide a comparison between the various models.

**Figure 8:**
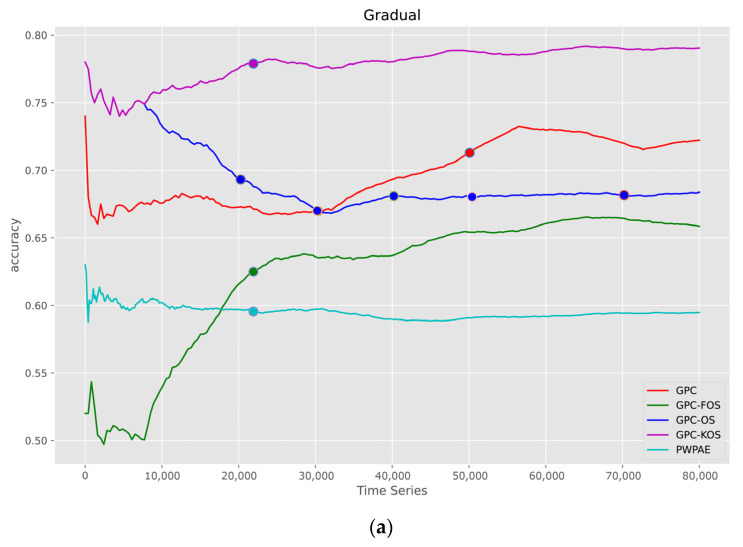

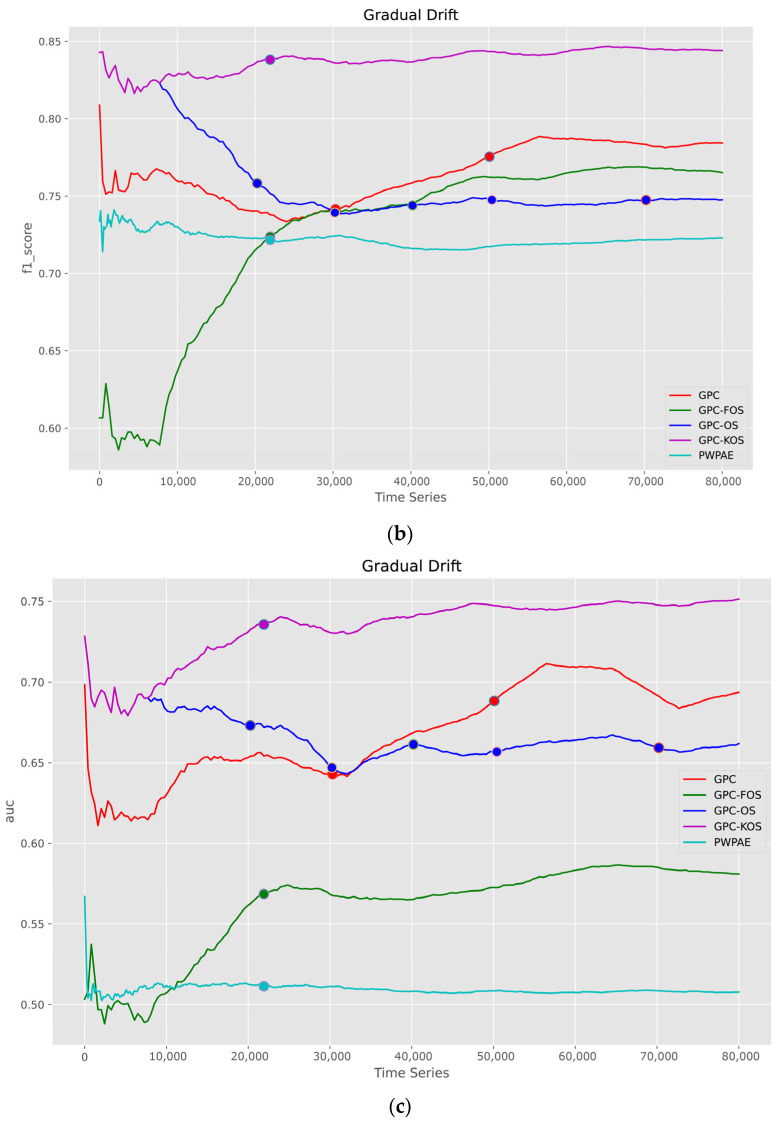
Visualisation of classification metrics as time series for a gradual type of drift—(**a**) accuracy, (**b**) F1-score, (**c**) AUC.

Figure 8 shows the evaluation of the performance generated by the models in the gradual variant of CD. It was observed that GPC-KOS, GPC-FOS, and GPC-OS outperformed the traditional GPC. In terms of accuracy, the traditional GPC started with a value of 0.74, while GPC-KOS and GPC-FOS started with 0.78. Additionally, it was found that the accuracy ended at a value of 0.79 for GPC-KOS compared to only 0.72 for the GPC. Furthermore, we observed that PWPAE had the least accurate performance compared to other models, with an accuracy of 0.6.

**Figure 9:**
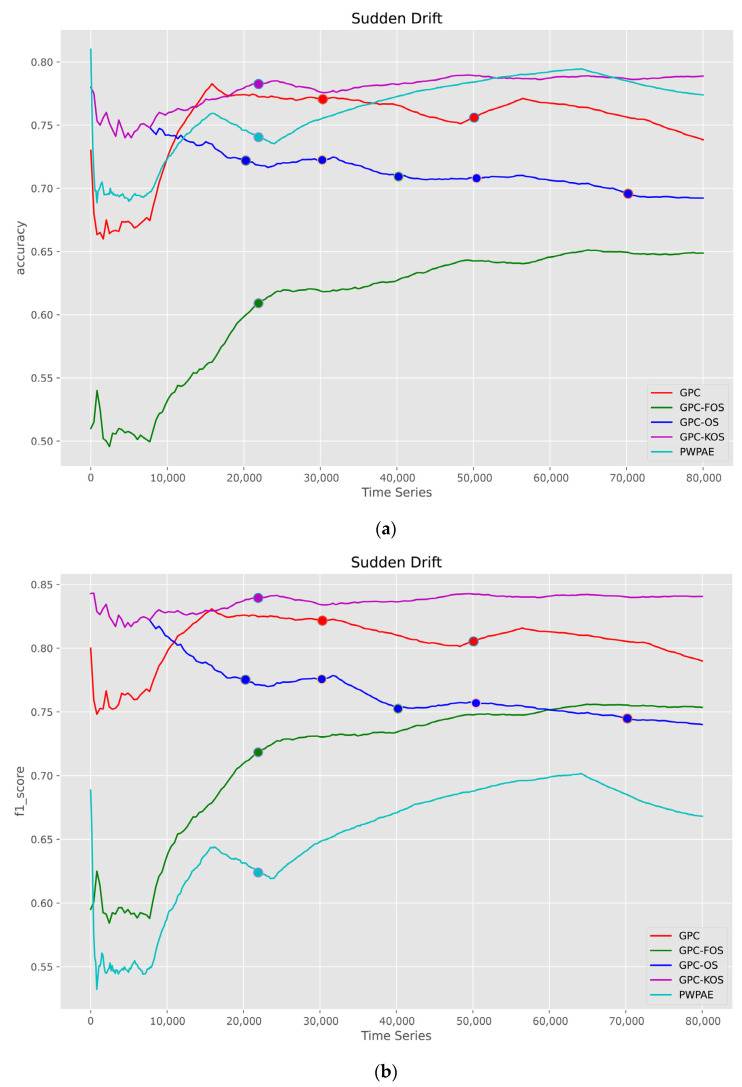

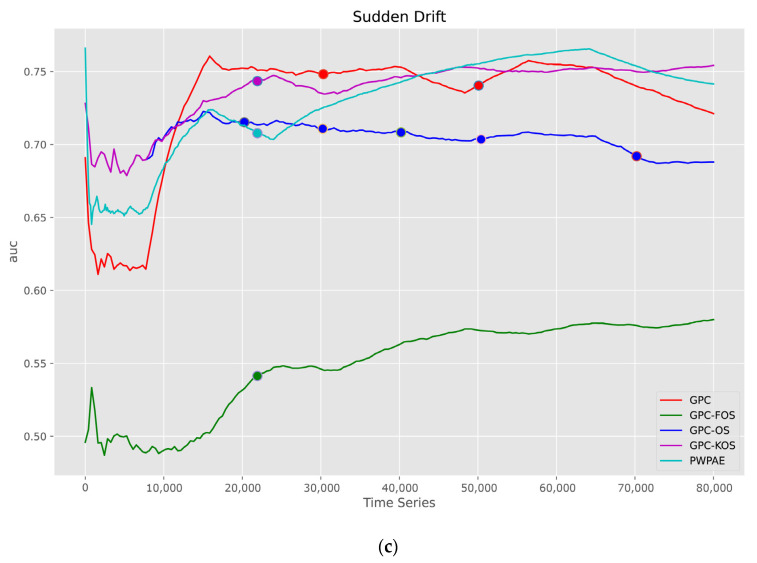
Visualisation of classification metrics as time series for a sudden type of drift—(**a**) accuracy, (**b**) F1-score, (**c**) AUC.

The second variant of CD was the sudden type, and its results are depicted in Figure 9. The results showed that GPC-KOS was superior in terms of accuracy. On the other hand, GPC-FOS had the lowest scoring performance in terms of accuracy and AUC. This suggested that upgrading the GPC to include learning transfer was not sufficient, while adding memory along with learning transfer was more beneficial. The results also showed that PWPAE had the lowest F1-score, indicating that its high accuracy was owing to its tendency to predict negative samples.

**Figure 10:**
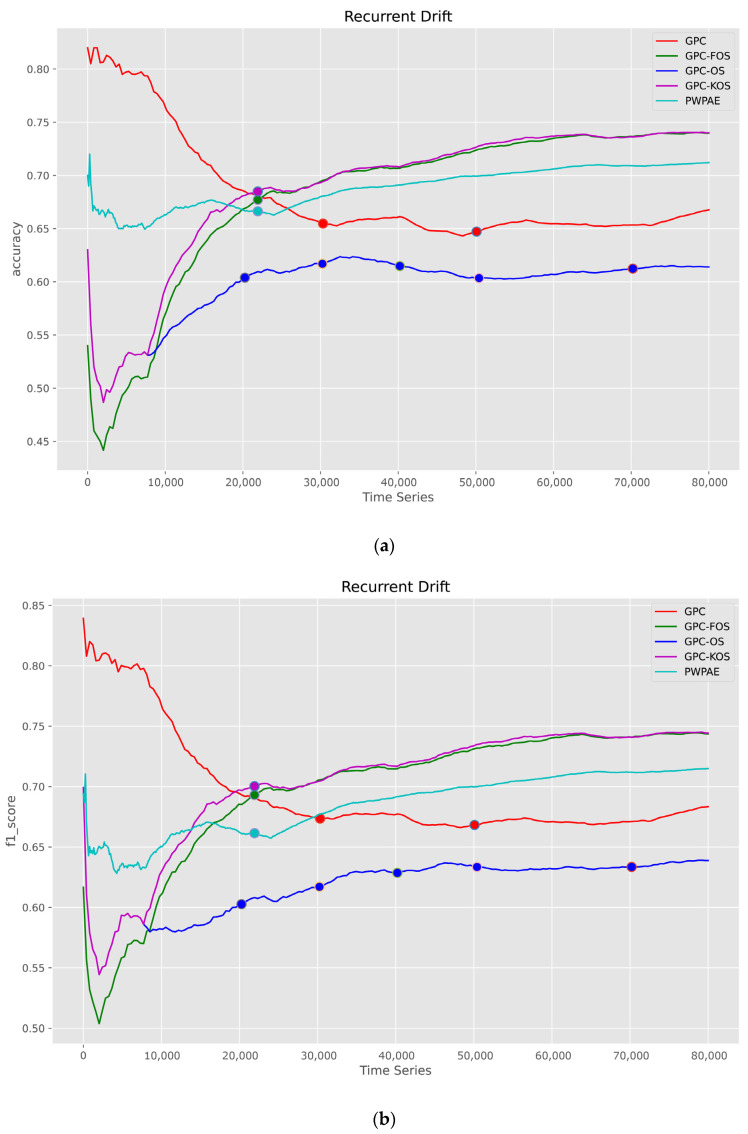

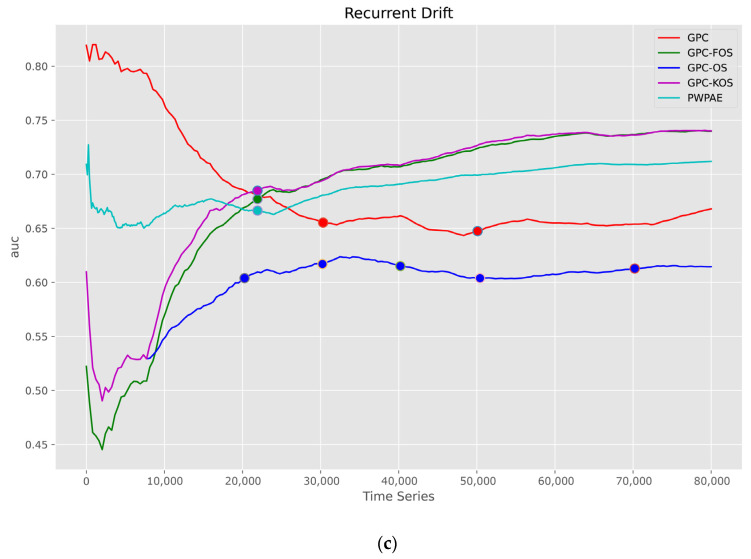
Visualisation of classification metrics as time series for a recurrent type of drift—(**a**) accuracy, (**b**) F1-score, (**c**) AUC.

The third variant of CD was the recurrent type, and its results are provided in Figure 10. We observed that the highest values of accuracy were stabilised by GPC-KOS and GPC-FOS at values of 0.74, which were superior to PWPAE, GPC, and GPC-OS. This suggested that the recurrent pattern of drift was more challenging for traditional methods such as the GPC and PWPAE. Additionally, the online nature of GPC-OS was not sufficient to deal with this type of drift, and it was crucial to have TL or memory to recall previous knowledge when it occurs again.

**Figure 11:**
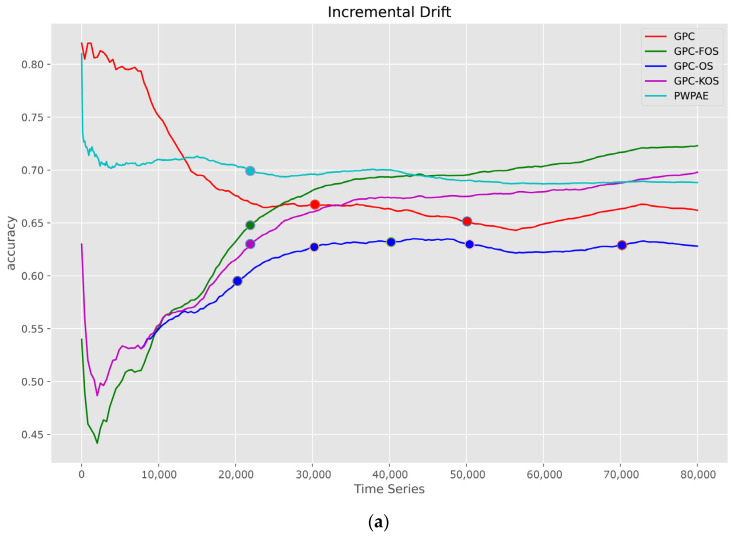

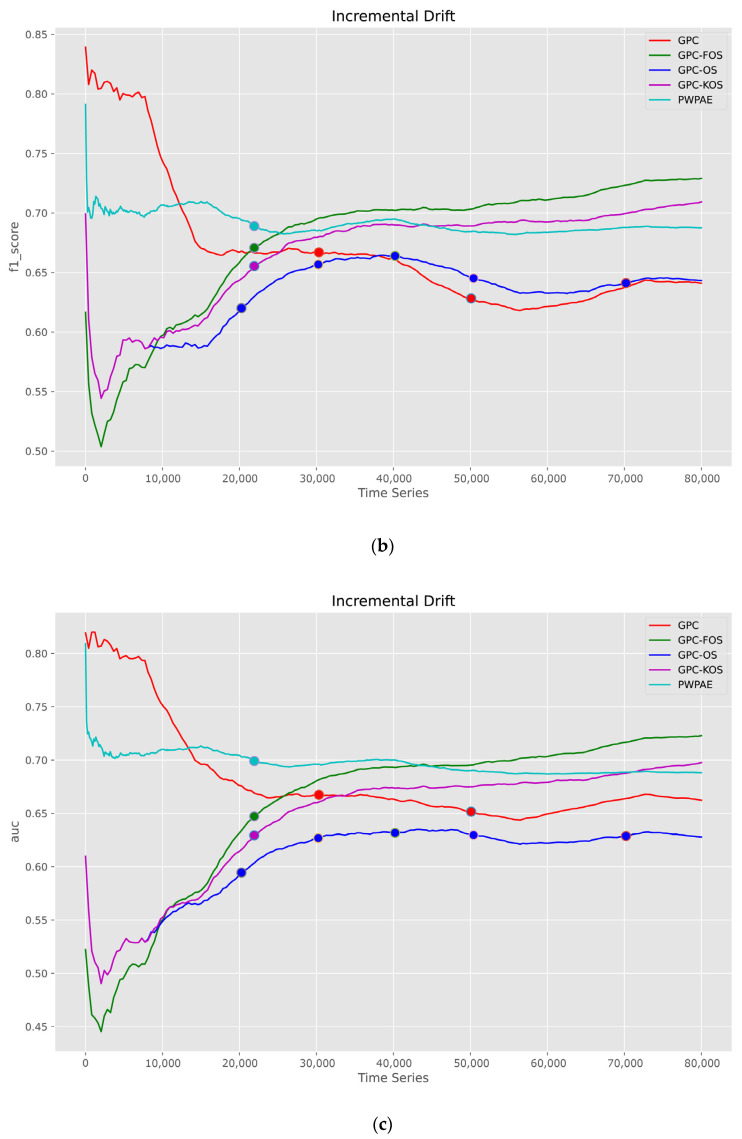
Visualisation of classification metrics as time series for an incremental type of drift—(**a**) accuracy, (**b**) F1-score, (**c**) AUC.

The time series of the performance metrics for incremental drift are presented in Figure 11. The results showed that GPC-FOS achieved the highest values for accuracy, F1-score, and AUC with a value of 0.73 for each.

**Figure 12:**
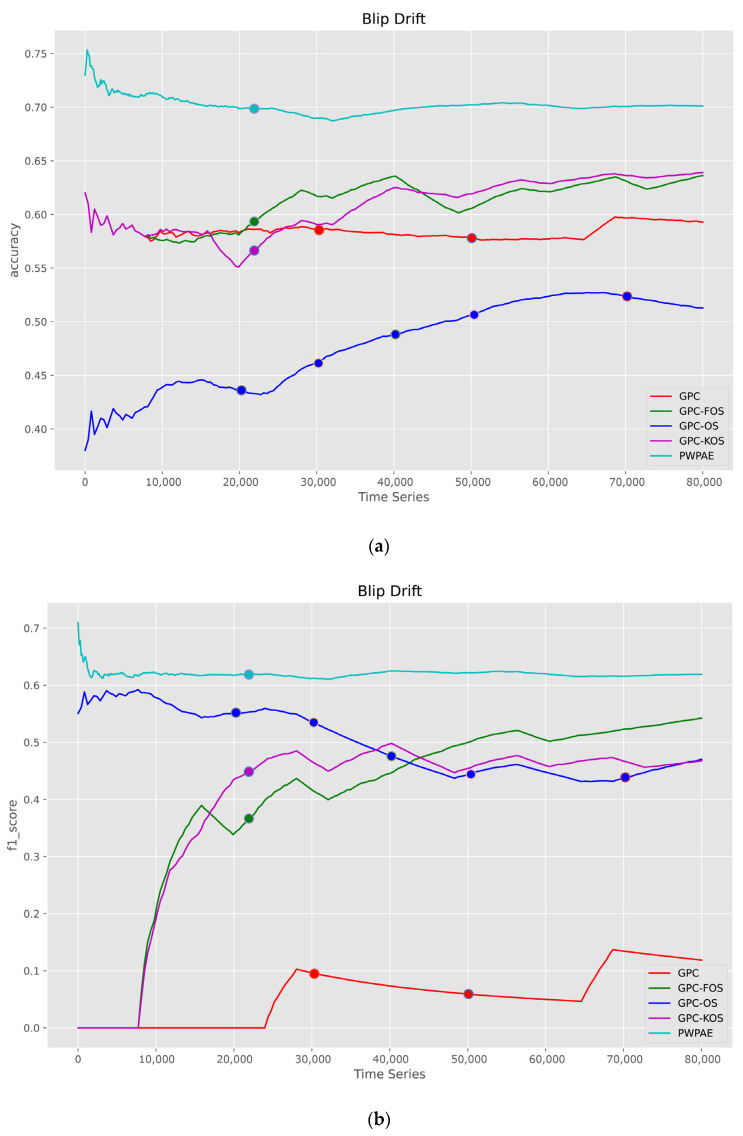

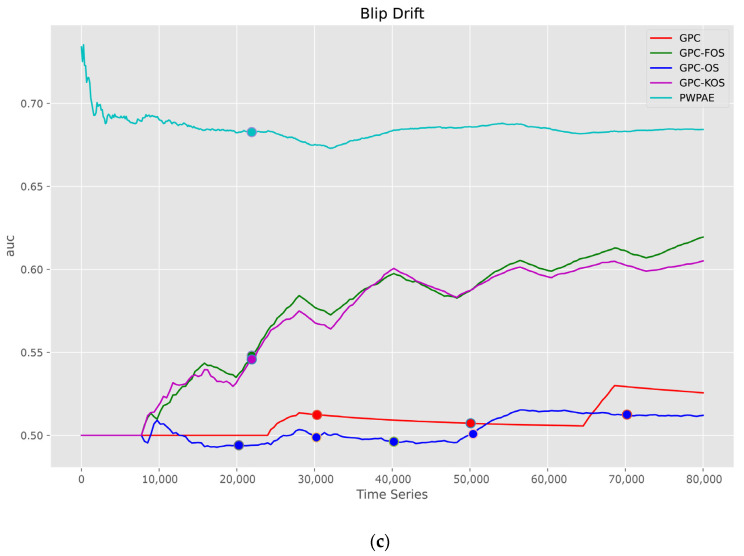
Visualisation of classification metrics as time series for a blip type of drift—(**a**) accuracy, (**b**) F1-score, (**c**) AUC.

According to the results of blip drift in Figure 12, it was found that the best performance was achieved by PWPAE with values of 0.70, 0.62, and 0.68 for accuracy, F1-score, and AUC, respectively. This revealed that the GPC family did not show the best performance for blip owing to the temporary change of pattern in blip drift and its short duration. Additionally, we found that both GPC-KOS and GPC-FOS were superior to the GPC and GPC-OS in this category.

### 5.2. Real-World Datasets IDS

This subsection presents the evaluation of our developed GPC models on real-world datasets, including CSE-CIC-IDS-2018, CICIDS-2017, ISCX2012, and KDD Cup ‘99, as well as overall results.

#### Overall Results

**Figure 13:**
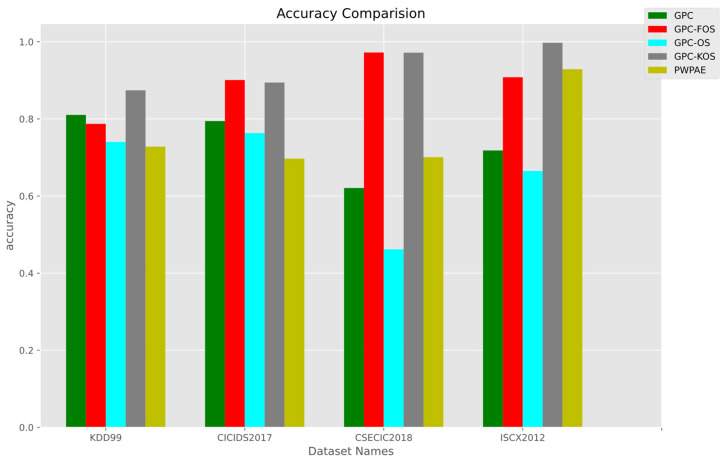
Accuracy of the incremental GPC (OS, FOS, KOS) variants and benchmarks (GPC, PWPAE) for real-world data.

**Figure 14:**
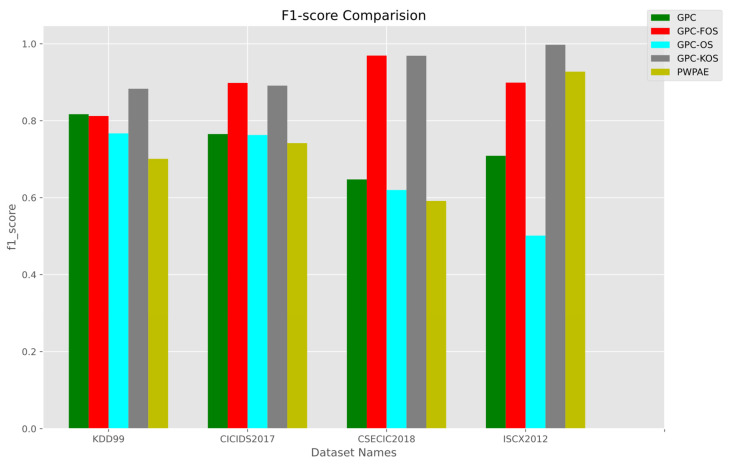
F1-score of the incremental GPC (OS, FOS, KOS) variants and benchmarks (GPC, PWPAE) for real-world data.

**Figure 15:**
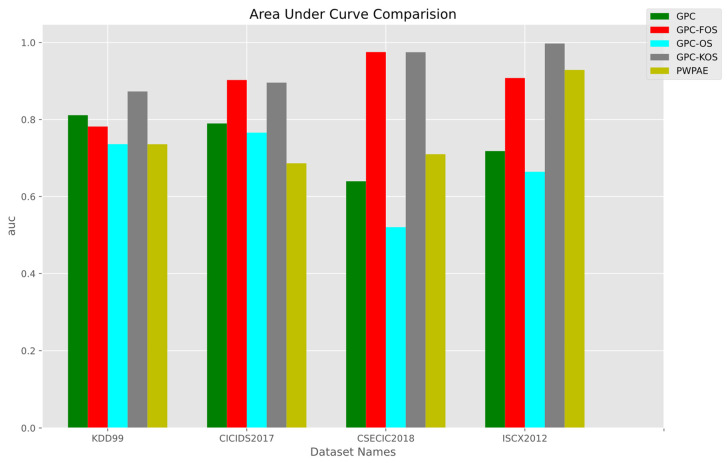
AUC of the incremental GPC (OS, FOS, KOS) variants and benchmarks (GPC, PWPAE) for real-world data.

The overall results are presented in Figure 13, Figure 14 and Figure 15, with a summary of numerical values in Table 7. It is noteworthy that GPC-FOS performed the best across all datasets, with competitive performance from GPC-KOS. The GPC-FOS scores for accuracy, F1-score, and AUC were 0.972249, 0.96934, and 0.975264, respectively. Similarly, the accuracy, F1-score, and AUC for GPC-KOS in CSE-CIC-IDS-2018 were 0.971836, 0.968905, and 0.974898, respectively. On the other hand, GPC-OS had the lowest performance with values of 0.461818, 0.619857, and 0.520479, respectively.

Moreover, regarding other datasets, GPC-KOS achieved the overall highest accuracy with values of 0.997481 for ISCX2012 and 0.997479 for F1-score. Additionally, PWPAE showed competitive performance to GPC-FOS only in ISCX2012 with an accuracy of 0.928886 for the former and 0.908099 for the latter, while GPC-KOS achieved an improved performance. Therefore, it was concluded that GPC-KOS and GPC-FOS were the methods providing the overall best performance across the considered datasets.

The proposed GPC framework was capable of handling new classes or categories of data as they arose since the incremental learning approach allowed for the addition of new classes by simply updating the classifiers with new data.

When a new class of data was encountered, the framework initially treated it as an outlier and trained the classifier to recognise it as such. As more examples of the new class were seen, the classifier gradually learned to recognise them as distinct classes and assign them separate labels. This process is called *incremental class learning* and allowed the framework to adapt to new categories of data in a flexible and efficient manner.

Moreover, the TL capability of the FA-OSELM and KP-OSELM classifiers in the GPC framework also helped in handling new classes. When a new class was added, the previously learned weights for relevant features were transferred to the new classifier, allowing it to learn more quickly and accurately. This approach helped to reduce the amount of data needed to train the new classifier and speeded up the learning process for new classes.

## 6. Advantages of the Incremental GPC

Our incremental GPC provides several advantages, which are as follows:

1. Improved performance in handling different CD variants: Our proposed framework provides novel strategies for handling different types of CD, such as incremental, sudden, recurrent, gradual, and blip. This results in improved classification performance compared to the traditional GPC and other state-of-the-art methods.
2. Memory features and TL: The proposed framework utilises TL and memory features, which allow the model to retain previous knowledge and adapt to incoming data. This results in improved performance in handling CD and reduces the need for retraining the model from scratch.
3. Enhanced data balancing and classifier update: The addition of data balancing and classifier update components to the GPC model further improves the accuracy of classification.
4. Real-world applicability: The proposed framework has been tested on real-world datasets, specifically in the context of a network IDS, which is a critical application in cybersecurity. This highlights the practical applicability of the proposed framework in real-world scenarios.
5. Comprehensive evaluation: Our work provides a comprehensive evaluation of the proposed framework on both synthetic and real-world datasets using standard classification metrics as time series, which allows for a fair and objective comparison against other state-of-the-art methods.

Overall, the proposed framework addresses the limitations of the traditional GPC in handling CD and provides novel strategies for achieving accurate classification in stream data scenarios.

## 7. Conclusions and Future Works

An intrusion detection system, particularly network IDS, is one application that makes use of stream data classification, which is a crucial ML task. Before classification, it involves sequentially updating knowledge. The most significant difficulty in stream data classification is CD, which denotes the alteration of the data’s statistical distribution over time. Different types of drift require different strategies. A core stream data classification method that handles both imbalance and CD is GPC classification [24].

However, two main drawbacks exist in the GPC that limit its performance with CD variants: its use of offline training classifiers, which causes performance degradation in online learning, and its lack of support for changes in active features, which causes knowledge loss. In this paper, we addressed these limitations by providing the first stream data-based classification framework that handles CD and its variants (sudden, incremental, gradual, recurrent, and blip). The framework provides various novel strategies for managing CD, including ensemble learning using the GPC, incremental learning using OSELM, and TL. We also introduced the usage of KP-OSELM to address certain drift variants that require restoring previous memory, such as sudden, recurrent, and gradual.

The evaluation was performed on both synthetic data and real-world data. The real-world data came from more than one field, with a focus on IDS. The experimental design considered comparisons between our approach and other state-of-the-art methods and included the generation of standard classification metrics of time series. Higher accuracy scores for gradual and sudden variants of drift were achieved by GPC-KOS, approximately 79%, while both GPC-KOS and GPC-FOS were equivalent and superior for recurrent variants, with an accuracy of 73%. On the other hand, the performance of both benchmarks (GPC and PWPAE) was inferior (66% and 59%, respectively) compared to our incremental variants of the GPC (GPC-KOS, GPC-OS, and GPC-FOS). In addition, despite our incremental variants of the GPC not achieving the best performance for blip drift, the overall performance of our proposal was competitive. Applying our incremental variants on real-world datasets revealed the superiority of GPC-FOS in both CSE-CIC-IDS-2018 and CICIDS-2017 datasets and GPC-KOS in both ISCX2012 and KDD Cup ‘99, with a maximum accuracy of 100% and 98% for GPC-KOS and GPC-FOS, respectively.

Finally, our contribution provides room for improvement in some specific instances, as described above. As future work, we plan to upgrade both GPC-KOS and GPC-FOS to handle blip drift in a more robust manner. Additionally, we suggest incorporating into our incremental GPC a functionality for handling feature evolution.

## Figures and Tables

**Table 1:** Overview of existing models for data stream prediction with an awareness and handling of CD for IDS application.

No	Article	Classifier	Incremental Learning	CD Detection Method	Dataset
1	[24]	Ensemble classifiers	×	Drift detection model (DDM) module analyses the data stream	ISCX IDS
2	[2]	Random Forest and Logistic Regression, SVM, K-means clustering, Ensemble	×	Sliding window-based K-means clustering	CIDDS-2017, NSL-KDD, Testbed datasets
3	[17]	INSOMNIA, a semi-supervised IDS	√	Incremental learning	CICIDS-2017
4	[25]	NCM classifiers	×	CD detection using auto encoder	Network and URL datasets
5	[20]	Adaptive Random Forest with Adaptive Windowing (ADWIN)	×	ADWIN	CIC-IDS 2018
6	[13]	K-nearest neighbour, SVM, Hoeffding Adaptive Tree, Adaptive Random Forest, SVM	×	ADWIN	KDD Cup ‘99
7	[26]	Extreme learning machine	×	×	KDD Cup ‘99, NSL-KDD Landsat
8	[1]	Linear-order algorithms and Gaussian-order algorithms	×	Online fusion of experts	NSL-KDD, ISCX
9	[27]	Reinforcement Learning	×	×	MAWIFlow
10	[28]	CNN and LSTM	×	Dynamic Residual Projection Method	Bot-IoT
11	[29]	Ensemble classifiers	×	ADWIN and EDDM	CICIDS-2017, IoTID ‘20
12	[30]	DNN	×	PCA-Based Drift Detection	traffic traces dataset
Our approach		OSELM, FA-OSELM and KP-OSELM	√	DDM	KDD Cup ’99, CICIDS-2017, CSE-CIC-IDS-2018, and ISCX2012

**Table 2:** Overview of existing models for data stream prediction with an awareness and handling of CD with the variants of drifts used for evaluation.

No	Article	Classifier	Application	Incremental Learning	CD Detection Method	Variants of CD Evaluated					Dataset
						Sudden	Incremental	Gradual	Recurrent	Blip	
13	[31]	Ensemble classifier	General	×	Generic scheme of drift detector	√	×	√	√	×	Wave, Sea, RBFGR, Tree R, Elec, Airline
14	[32]	SVM	General	×	ADWIN	√	×	√	×	×	SINE1, CIRCLES, COVERTYPE
15	[33]	Ensemble classifier	General	×	Not specified	√	×	√	√	×	KDD Cup ‘99, Sensor Data, Hyperplane Stagger LED, SEA, Airlines, Cover type
16	[34]	Random forests	General	×	Not specified	√	×	√	√	×	Electricity Rain, Airlines Covtype, Sensor
17	[35]	Recurrent drift classifier	General	×	Proactive Drift Detection	√	×	√	×	×	Agrawal, Random Tree, data generators
18	[36]	Ensemble classifiers	General	×	Clustering in the Model Space (CDCMS)	√	×	√	√	×	KDD Cup ‘99, Sensor, Power Supply, Sine1, Sine2, Agr1 Agr4, SEA1, SEA2, STA1, STA2
19	[8]	Heterogeneous ensemble classifiers (Naive Bayes, k-NN, decision trees)	General	×	Not specified	√	×	√	×	×	KDD Cup ‘99, ELEC, Airlines, AIRL, COVT MIXEDBALANCED WAVEFORM, RBF
20	[37]	Ensemble classifiers	General	×	Kappa Updated Ensemble (KUE)	√	×	√	√	×	RBF, Random Tree, Agrawal, Asset Negotiation, LED, Hyperplane, Mixed, SEA, Sine, STAGGER, Waveform Power supply Electricity
21	[38]	Ensemble classifiers	General	×	Major accuracy decline	√	×	√	√	×	Spam, Electricity, Email, Phishing, Poker, Usenet, Weather, Interchanging RBF, MG2C2D, Moving Squares, Avg. Rank
22	[39]	Ensemble classifiers	General	×	ADWIN, DDM	√	√	√	√	×	IoTID20 and CICIDS-2017
23	[40]	Elastic gradient boosting decision tree (eGBDT)	General	×	Adaptive iterations (AdIter)	√	√	×	×	×	Electricity, Weather, Spam, Usenet1, Usenet2, Airline, SEAa, RTG, RBF, HYP
Our approach		OSELM, FA-OSELM and KP-OSELM	IDS	√	DDM	√	√	√	√	√	KDD Cup ’99, CICIDS-2017, CSE-CIC-IDS-2018, and ISCX2012

**Table 3:** Symbols and their descriptions.

Symbol	Description	Symbol	Description
N	The number of inputs	*H*	Output matrix
$x_{i}$	The record of features	*T*	Target vectors
$t_{i}$	The target of certain record	*m*	Number of features
i	An index	*k*	Index
$b_{i}$	Biases	$M_{k}$	Intermediate matrix
*g*(*x*)	An activation function	RLS	Recursive least square
$N_{h}$	The number of neurons	$w_{i}$	Weights between input hidden layer
β	Weights between hidden output layer	C	Regularisation parameter

**Table 4:** Parameters of generating data stream from MOA.

Parameter	Value
No. of samples	80,000
Chunk size	8000
Test percentage	0.8
No. of Features of Gradual	4
No. of Features of Recurrent Drift	11
No. of Features of Blip	16
No. of Features of Incremental Drift	11
No. of Features of Sudden	4

**Table 5:** A statistical overview of the real-world datasets.

No	Dataset	Number of Features	Number of Instances	Name of Attacks
1	KDD Cup ‘99	42	4,898,431	Dos, Probe, R2L, and U2R.
2	CICIDS-2017	79	2,830,743	DoS Hulk, PortScan, DDoS, DoS GoldenEye, FTP-Patator, SSHPatator, DoS slowloris, DoS Slowhttptest, Bot, Web Attack—Brute Force, Web Attack—XSS, Infiltration, Web Attack—Sql Injection, Heartbleed.
3	CSE-CIC-IDS-2018	80	4,525,399	Bot, Brute Force, Dos, Infiltration, SQL injection.
4	ISCX2012	19	2,545,935	Infiltrating, Brute force SSH, HTTP denial of service (DoS), and Distributed Denial of service (DDoS).

**Table 6:** Summary of numerical values of accuracy, F1-score, and AUC for GPC variants (OS, FOS, KOS) and benchmarks in synthetic data.

**Gradual**					
	**GPC-KOS**	**GPC** [24]	**GPC-FOS**	**GPC-OS**	**PWPAE** [39]
Accuracy	**0.790500**	0.722650	0.658500	0.683700	0.594899
F1_score	**0.844099**	0.784641	0.765340	0.747526	0.723136
AUC	**0.751566**	0.694150	0.580759	0.661976	0.507830
**Sudden**					
	**GPC-KOS**	**GPC** [24]	**GPC-FOS**	**GPC-OS**	**PWPAE** [39]
Accuracy	**0.788550**	0.736850	0.649300	0.692200	0.773712
F1_score	**0.840469**	0.788439	0.753877	0.739770	0.667857
AUC	**0.753872**	0.719807	0.580746	0.6877985	0.741358
**Recurrent**					
	**GPC-KOS**	**GPC-FOS**	**PWPAE** [39]	**GPC** [24]	**GPC-OS**
Accuracy	0.739550	**0.739600**	0.711919	0.668550	0.613600
F1_score	**0.743891**	0.743524	0.715082	0.684078	0.638946
AUC	0.739689	**0.739728**	0.711876	0.668892	0.614074
**Incremental**					
	**GPC-FOS**	**GPC-KOS**	**PWPAE** [39]	**GPC-OS**	**GPC** [24]
Accuracy	**0.723250**	0.698850	0.688198	0.627600	0.660750
F1_score	**0.7298088**	0.709945	0.687453	0.642953	0.639919
AUC	**0.723171**	0.698698	0.688199	0.627427	0.661011
**Blip**					
	**PWPAE** [39]	**GPC-FOS**	**GPC-OS**	**GPC-KOS**	**GPC** [24]
Accuracy	**0.701134**	0.637100	0.511950	0.640050	0.592750
F1_score	**0.618994**	0.542832	0.472179	0.469452	0.117647
AUC	**0.684335**	0.620218	0.511852	0.606036	0.525374

**Table 7:** Summary of numerical values of accuracy, F1-score, and AUC for GPC variants (OS, FOS, KOS) and benchmarks for real-world data.

**CSE-CIC-IDS-2018**					
	**GPC-FOS**	**GPC-KOS**	**GPC** [24]	**GPC-OS**	**PWPAE** [39]
Accuracy	**0.972249**	0.971836	0.620854	0.461818	0.700703
F1_score	**0.969346**	0.968905	0.647431	0.619857	0.591503
auc	**0.975264**	0.974898	0.639654	0.520479	0.709977
**CICIDS-2017**					
	**GPC-FOS**	**GPC-KOS**	**GPC** [24]	**GPC-OS**	**PWPAE** [39]
Accuracy	**0.900965**	0.894352	0.794415	0.763269	0.697047
F1_score	**0.898320**	0.891201	0.765363	0.762805	0.741752
AUC	**0.902722**	0.895905	0.789806	0.765984	0.686410
**ISCX2012**					
	**GPC-KOS**	**PWPAE** [39]	**GPC-FOS**	**GPC** [24]	**GPC-OS**
Accuracy	**0.997481**	0.928886	0.908099	0.718229	0.665100
F1_score	**0.997479**	0.927572	0.898989	0.708829	0.501344
AUC	**0.997485**	0.928850	0.907880	0.718153	0.664284
**KDD Cup ‘99**					
	**GPC-KOS**	**GPC** [24]	**GPC-FOS**	**GPC-OS**	**PWPAE** [39]
Accuracy	**0.874269**	0.810431	0.787163	0.740344	0.728109
F1_score	**0.883099**	0.816953	0.812278	0.767096	0.700947
AUC	**0.872926**	0.811310	0.781891	0.736063	0.735894

## Data Availability

Not applicable.

## References

[1] Yazdi H.S., Bafghi A.G. (2020). A drift aware adaptive method based on minimum uncertainty for anomaly detection in social networking. Expert Syst. Appl..

[2] Jain M., Kaur G. (2021). Distributed anomaly detection using concept drift detection based hybrid ensemble techniques in streamed network data. Clust. Comput..

[3] Zhang Z., Chen L., Liu Q., Wang P. (2020). A fraud detection method for low-frequency transaction. IEEE Access.

[4] Mansour R.F., Al-Otaibi S., Al-Rasheed A., Aljuaid H., Pustokhina I., Pustokhin D.A. (2021). An optimal big data analytics with concept drift detection on high-dimensional streaming data. CMC Comput. Mater. Contin..

[5] Neto A.F., Canuto A.M. (2021). EOCD: An ensemble optimization approach for concept drift applications. Inf. Sci..

[6] Wang S., Minku L.L., Yao X. (2018). A systematic study of online class imbalance learning with concept drift. IEEE Trans. Neural Netw. Learn. Syst..

[7] Kalid S.N., Ng K.-H., Tong G.-K., Khor K.-C. (2020). A multiple classifiers system for anomaly detection in credit card data with unbalanced and overlapped classes. IEEE Access.

[8] Sarnovsky M., Kolarik M. (2021). Classification of the drifting data streams using heterogeneous diversified dynamic class-weighted ensemble. Peer J. Comput. Sci..

[9] Chi H.R., Wu C.K., Huang N.-F., Tsang K.F., Radwan A. (2022). A Survey of Network Automation for Industrial Internet-of-Things Towards Industry 5.0. IEEE Trans. Ind. Inform..

[10] Leng J., Sha W., Wang B., Zheng P., Zhuang C., Liu Q., Wuest T., Mourtzis D., Wang L. (2022). Industry 5.0: Prospect and retrospect. J. Manuf. Syst..

[11] Demir K.A., Döven G., Sezen B. (2019). Industry 5.0 and human-robot co-working. Procedia Comput. Sci..

[12] Angelopoulos A., Michailidis E.T., Nomikos N., Trakadas P., Hatziefremidis A., Voliotis S., Zahariadis T. (2019). Tackling faults in the industry 4.0 era—A survey of machine-learning solutions and key aspects. Sensors.

[13] Martindale N., Ismail M., Talbert D.A. (2020). Ensemble-based online machine learning algorithms for network intrusion detection systems using streaming data. Information.

[14] Adnan A., Muhammed A., Abd Ghani A.A., Abdullah A., Hakim F. (2021). An intrusion detection system for the internet of things based on machine learning: Review and challenges. Symmetry.

[15] Jain M., Kaur G., Saxena V. (2022). A K-Means clustering and SVM based hybrid concept drift detection technique for network anomaly detection. Expert Syst. Appl..

[16] Folino F., Folino G., Guarascio M., Pisani F.S., Pontieri L. (2021). On learning effective ensembles of deep neural networks for intrusion detection. Inf. Fusion.

[17] Andresini G., Pendlebury F., Pierazzi F., Loglisci C., Appice A., Cavallaro L. Insomnia: Towards concept-drift robustness in network intrusion detection. Proceedings of the 14th ACM Workshop on Artificial Intelligence and Security.

[18] Lu J., Liu A., Dong F., Gu F., Gama J., Zhang G. (2019). Learning under Concept Drift: A Review. IEEE Trans. Knowl. Data Eng..

[19] Guo H., Li H., Ren Q., Wang W. (2022). Concept drift type identification based on multi-sliding windows. Inf. Sci..

[20] Seth S., Singh G., Chahal K. Drift-based approach for evolving data stream classification in Intrusion detection system. Proceedings of the Workshop on Computer Networks & Communications.

[21] Liu Q., Wang D., Jia Y., Luo S., Wang C. (2022). A multi-task based deep learning approach for intrusion detection. Knowl.-Based Syst..

[22] Zhou Y., Mazzuchi T., Sarkani S. (2020). M-AdaBoost-A based ensemble system for network intrusion detection. Expert Syst. Appl..

[23] Han B.-H., Li Y.-M., Liu J., Geng S.-L., Li H.-Y. (2014). Elicitation criterions for restricted intersection of two incomplete soft sets. Knowl.-Based Syst..

[24] Folino G., Pisani F.S., Pontieri L. (2020). A GP-based ensemble classification framework for time-changing streams of intrusion detection data. Soft Comput..

[25] Kuppa A., Le-Khac N.-A. (2022). Learn to adapt: Robust drift detection in security domain. Comput. Electr. Eng..

[26] Adnan A., Muhammed A., Abd Ghani A.A., Abdullah A., Hakim F. (2020). Hyper-heuristic framework for sequential semi-supervised classification based on core clustering. Symmetry.

[27] dos Santos R.R., Viegas E.K., Santin A.O., Cogo V.V. (2022). Reinforcement learning for intrusion detection: More model longness and fewer updates. IEEE Trans. Netw. Serv. Manag..

[28] Qiao H., Novikov B., Blech J.O. (2021). Concept Drift Analysis by Dynamic Residual Projection for effectively Detecting Botnet Cyber-attacks in IoT scenarios. IEEE Trans. Ind. Inform..

[29] Yang L., Shami A. (2022). A Multi-Stage Automated Online Network Data Stream Analytics Framework for IIoT Systems. IEEE Trans. Ind. Inform..

[30] Wahab O.A. (2022). Intrusion detection in the iot under data and concept drifts: Online deep learning approach. IEEE Internet Things J..

[31] Mahdi O.A., Pardede E., Ali N. (2021). A hybrid block-based ensemble framework for the multi-class problem to react to different types of drifts. Clust. Comput..

[32] Gâlmeanu H., Andonie R. (2021). Concept Drift Adaptation with Incremental–Decremental SVM. Appl. Sci..

[33] Museba T., Nelwamondo F., Ouahada K., Akinola A. (2021). Recurrent adaptive classifier ensemble for handling recurring concept drifts. Appl. Comput. Intell. Soft Comput..

[34] Wu O., Koh Y.S., Dobbie G., Lacombe T. (2022). Probabilistic exact adaptive random forest for recurrent concepts in data streams. Int. J. Data Sci. Anal..

[35] Wu O., Koh Y.S., Dobbie G., Lacombe T. Nacre: Proactive recurrent concept drift detection in data streams. Proceedings of the 2021 International Joint Conference on Neural Networks (IJCNN).

[36] Chiu C.W., Minku L.L. (2020). A diversity framework for dealing with multiple types of concept drift based on clustering in the model space. IEEE Trans. Neural Netw. Learn. Syst..

[37] Cano A., Krawczyk B. (2020). Kappa updated ensemble for drifting data stream mining. Mach. Learn..

[38] Bakhshi S., Ghahramanian P., Bonab H., Can F. (2021). A Broad Ensemble Learning System for Drifting Stream Classification. arXiv.

[39] Yang L., Manias D.M., Shami A. (2021). PWPAE: An Ensemble Framework for Concept Drift Adaptation in IoT Data Streams. Proceedings of the 2021 IEEE Global Communications Conference (GLOBECOM).

[40] Wang K., Lu J., Liu A., Song Y., Xiong L., Zhang G. (2022). Elastic gradient boosting decision tree with adaptive iterations for concept drift adaptation. Neurocomputing.

[41] Huang G.-B., Liang N.-Y., Rong H.-J., Saratchandran P., Sundararajan N. (2005). On-line sequential extreme learning machine. Comput. Intell..

[42] Jiang X., Liu J., Chen Y., Liu D., Gu Y., Chen Z. (2016). Feature adaptive online sequential extreme learning machine for lifelong indoor localization. Neural Comput. Appl..

[43] Al-Khaleefa A., Ahmad M., Isa A., Esa M.R.M., Aljeroudi Y., Jubair M.A., Malik R.F. (2019). Knowledge preserving OSELM model for Wi-Fi-based indoor localization. Sensors.

[44] Al-Khaleefa A.S., Ahmad M.R., Isa A.A.M., Esa M.R.M., Al-Saffar A., Aljeroudi Y. (2018). Infinite-Term Memory Classifier for Wi-Fi Localization Based on Dynamic Wi-Fi Simulator. IEEE Access.

[45] Tavallaee M., Bagheri E., Lu W., Ghorbani A.A. (2009). A detailed analysis of the KDD CUP 99 data set. Proceedings of the 2009 IEEE Symposium on Computational Intelligence for Security and Defense Applications.

[46] Sharafaldin I., Lashkari A.H., Ghorbani A.A. (2018). Toward generating a new intrusion detection dataset and intrusion traffic characterization. ICISSp.

[47] Shiravi A., Shiravi H., Tavallaee M., Ghorbani A.A. (2012). Toward developing a systematic approach to generate benchmark datasets for intrusion detection. Comput. Secur..

